# Stochastic dominance spanning and augmenting the human development index with institutional quality

**DOI:** 10.1007/s10479-022-04656-w

**Published:** 2022-04-13

**Authors:** Mehmet Pinar, Thanasis Stengos, Nikolas Topaloglou

**Affiliations:** 1 Business School, Edge Hill University, St Helens Road, Ormskirk, L39 4QP Lancashire UK; 2 Department of Economics, University of Guelph, Guelph, ON N1G 2W1 Canada; 3 IPAG Business School, 75005 Paris, France; 4 Department of International European and Economic Studies, AUEB, 76, Patision Street, 10434 Athens, Greece

**Keywords:** Well-being, Human development, Institutions, Stochastic dominance, C14, C61, D81, I31, O15

## Abstract

The well-known Human Development Index (HDI) goes beyond a single measure of well-being as it is constructed as a composite index of achievements in education, income, and health dimensions. However, it is argued that the above dimensions do not reflect the overall well-being, and new indicators should be included in its construction. This paper uses stochastic dominance spanning to test the inclusion of additional institutional quality (governance) dimensions to the HDI, and we examine whether the augmentation of the original set of welfare dimensions by an additional component leads to distributional welfare gains or losses or neither. We find that differently constructed indicators of the same institutional quality measure produce different distributions of well-being.

**Supplementary Information:**

The online version contains supplementary material available at 10.1007/s10479-022-04656-w.

## Introduction

Well-being is inherently a multidimensional concept (see, e.g., Fleurbaey, 2009; Fleurbaey & Blanchet, 2013) and there is an extensive list of composite indices that have been developed to measure the overall multidimensional welfare progress for various groups of countries (see Singh et al., 2012 for a recent overview of a large set of composite indices). The Human Development Index (HDI) is one of the most-known composite indices that measures the average achievement of countries in education, life expectancy, and GNI per capita (Malik, 2013), which goes beyond the single measurement of well-being.

Even though composite indices serve to convey a quick message to stakeholders and policymakers about the strengths and weaknesses of current policies that promote development (Ness et al., 2007), they rely on value judgments (i.e., selection of the indicators, normalization of indicators, and weight allocation to indicators) and as such, they have drawn criticisms in the literature (see Decancq & Lugo, 2013 for a comprehensive discussion on the topic). For instance, the construction of HDI received many criticisms regarding the selection of its constituent indicators (Bravo, 2014; Ranis et al., 2006; Stiglitz et al., 2009), normalization’s effect on composite scores (see e.g., Noorbakhsh, 1998; Pollesch & Dale, 2016), and weight choices in the aggregation procedure (Athanassoglou, 2015; Cherchye et al., 2007, 2008; Foster et al., 2013; Mahlberg & Obersteiner, 2001; Mizobuchi, 2014; Permanyer, 2011; Pinar et al., 2013; Tofallis, 2013; Zanella et al., 2015; among many others).^1^

This paper aims at contributing to this literature. It has been argued that HDI only captures well-being achievements in three dimensions (i.e., education, health and standard of living) and new indicators should be included to the component list (e.g., Ranis et al., 2006 suggested inclusion of more indicators to the already existing set of indicators; Bravo, 2014 analyzed the inclusion of environmental dimension to the HDI; Stiglitz et al. (2009) identified eight dimensions to measure multidimensional well-being). However, if a new set of indicators is to be included in the component list of HDI, one needs to provide an answer to the following questions: (1) which well-being dimension should be included in the component list of HDI? (2) what set of proxies can be used to measure the included well-being dimension and which one to choose to measure the augmented HDI? (3) after the inclusion of the new well-being concept to the indicator list, what set of weights are to be given to each dimension? Our paper aims at providing answers to these questions. First, in the next section, we provide a discussion on why the inclusion of governance to the indicator list of HDI is worthwhile. Second, there are many proxies available to measure the quality of governance and also many weight alternatives to choose from to obtain an augmented index. This paper offers a new methodology that can be considered as an assessment tool to test the inclusion of indicators with admissible weights (i.e., the weights allocated to four dimensions are allowed to vary between lower and upper bounds) offering a way to compare many available options (i.e., many proxies and weights to choose from).

Our paper provides a data-driven methodology that allows for the possible inclusion of additional well-being components to HDI based on stochastic dominance (SD) spanning analysis. More specifically, this paper will examine whether the inclusion of a given governance indicator to the already existing composite HDI leads to distributional gains (or losses) when weights are allowed to vary within the admissible range. The comparison of the empirical achievement distributions with and without the included indicator will be made using SD spanning. The latter extends the SD efficiency methodology to test whether the inclusion of an indicator to the existing composite index (i.e., HDI) leads to distributional welfare gains or losses or neither.

SD is a powerful framework of analysis that has been used in a wide variety of applications in economics, finance, and statistics (see, e.g., Levy, 2015, for an overview and references). Due to its non-parametric attractiveness, SD is particularly appealing for comparisons of variables with asymmetric profiles (e.g., income, life expectancy, human capital distributions among different countries). There is an extensive literature regarding the use of SD concepts for pairwise welfare comparisons (Atkinson, 1987; Barrett & Donald, 2003; Davidson & Duclos, 2000; Duclos et al., 2005, 2008; Ferre et al., 2012; Foster & Shorrocks, 1988; Gonzalo & Olmo, 2014; Linton et al., 2005; Maasoumi, 1986; Van de gaer et al., 2014)) and multivariate ones (see, e.g., Anderson & Post, 2018 Anderson et al., 2020; Bennett & Mitra, 2013; Consigli et al., 2020; Duclos et al., 2006a, 2006b and 2011; Kopa et al., 2018; Maasoumi & Racine, 2016; Sharma & Mehra, 2017; Vitali & Moriggia, 2021; Yalonetzky, 2013).

In a related literature in finance, a more general, multivariate problem is that of testing whether a given portfolio is stochastically efficient relative to all mixtures of a discrete set of alternatives (Kuosmanen, 2004; Post, 2003; Roman et al., 2006), while others address this problem with various proposed SD efficiency (SDE) tests (Post & Versijp, 2007; Scaillet & Topaloglou, 2010; Lizyayev, 2012; Linton et al., 2014; Arvanitis & Topaloglou, 2017; Fang & Post, 2017 and Post & Poti, 2017). These SDE tests are used to examine the existence of alternative ways of combining assets that dominate the benchmark market or welfare index to obtain best- and worst-case scenarios of well-being (e.g., Pinar et al., 2013; Agliardi et al., 2015; Pinar et al., 2015; Pinar et al., 2017; Mehdi, 2019) and risk indices (see e.g., Agliardi et al., 2012, 2014). Furthermore, recent studies also obtain forecast combinations that offered a lower expected loss than other forecast combinations for all permissible loss functions (see e.g., Jin et al., 2017; Post et al., 2019), and for different quantiles of the expected loss function (see e.g., Corradi & Swanson, 2013; Pinar et al., 2018).

The papers mentioned above use a given set of assets (well-being dimensions) to examine whether a given portfolio is efficient (well-being index produces the best or worst-case scenario), but they were not designed for testing the inclusion of additional assets (indicators) to the existing portfolio (welfare index). The concept of SD spanning has been recently introduced by Arvanitis et al. (2019) as a model-free alternative to mean–variance spanning (Huberman & Kandel, 1987). In a finance setting, Arvanitis et al. (2019) examine whether the inclusion of new assets leads to distributional gains to the market portfolio or not. In the context of additional dimension inclusion to the HDI, this paper evaluates whether SD spanning occurs if there are no gains or losses from a particular expansion of the given feasible choice set (i.e., income, life expectancy, education). The null hypothesis in the SD spanning testing framework is that the introduction of a new component of human development *does not* lead to distributional welfare gains (or losses). On the other hand, if the inclusion of an indicator leads to a welfare-improving or -deteriorating augmentation of the choice set of indicators, this indicator’s inclusion would imply a rejection of the null hypothesis as the original set would not form a spanning set. In a recent paper, Arvanitis et al. (2021) proposed stochastic bounds, which complements the concept of stochastic spanning (Arvanitis et al., 2019). The SD spanning used in this paper does not require a combination of the original set of indicators to dominate all the welfare indices in the extended set of indicators. However, the stochastic bounds concept would allow one to identify a combination of original indicators that would offer higher measured wellbeing by identifying a specific weighting scheme that dominates a set of alternatives. Since this paper aims to test the inclusion of a given indicator to the HDI indicator list, and not to identify the weighting scheme that dominates a set of alternatives, we use SD spanning methodology in this paper.

Our paper deviates from the SD spanning tests used for portfolio choice applications in two ways. First, we not only test for SD spanning for the scenario of welfare gains but also for welfare losses. Second, we impose a minimum weight to the well-being dimensions since most decision-makers prefer to avoid corner solutions to preserve the multidimensional nature of well-being (see e.g., Tone & Tsutsui, 2010; Foster et al., 2013; Seth & McGillivray, 2018; Pinar et al., 2020 for the use of reasonable weights for measuring multidimensional well-being and/or poverty). Furthermore, since there are no universally agreed weights on well-being dimensions and any valid choice of weights could be somehow justified depending on the specific context as it may be politically driven (see, e.g., Ravallion, 2011), instead of choosing and trying some of the many alternative possible weights given to well-being dimensions, we only set a minimum acceptable weight to test whether the inclusion of governance proxies lead to further distributional welfare gains or losses or neither.

The remaining part of the paper is organized as follows. Section 2 provides a discussion on why the inclusion of governance as part of the composite development index is conceptually a good way forward. Section 3 presents the stochastic spanning framework. Section 4 presents the data, and the results obtained with the empirical application of the SD spanning methodology. Finally, Sect. 5 concludes. The appendix also presents the statistical theory underlying the stochastic spanning tests and the computational strategy for the test statistic.

## Governance and well-being

There has been an extensive discussion on the importance of governance that links it to Sen’s capability approach (Sen, 1985, 1987, 1999). It has been argued that the quality of the legal system and the presence of political rights could foster freedom of thought and political participation that would improve the capabilities of individuals (see, e.g., Robeyns, 2005 for detailed discussion and survey on social and formal institutions’ relationship with the capabilities approach). Stiglitz et al. (2009) highlight the importance of political voice and governance (concepts that are closely linked with institutional quality) in shaping freedom of choice and speech and that better rule of law and legislative guarantees would enhance the quality of life of all citizens (see Sect. 4.4 of the report for further discussion on how political voice and governance reinforce better quality of life for citizens). On the other hand, Feldmann (2017) show that the economic freedom also promotes human capital investment (see also Graafland, 2020 for the positive relationship between economic freedom and human development). Finally, the importance of governance has also been emphasized by the United Nations Assembly (2013), and it has been pointed out that the “implementation of a post-2015 development agenda will depend, critically, on effective governance capacities” (p. 33, UN System Task Team on the Post-2015 UN Development Agenda, 2012a).^2^

Beyond the conceptual importance of governance, there exists an extensive empirical literature that identifies its importance for social and economic outcomes. For instance, it has been long argued that the quality of institutions is one of the main factors that explain the long-term income differences across countries (see e.g., Acemoglu et al., 2001, 2014; Easterly & Levine, 2003; Rodrik et al., 2004; Bosker & Garretsen, 2009; Pinar, 2015 among many others). In a seminal paper, Acemoglu et al. (2001) argued that the hostile environment of new diseases faced by the European settlers promoted different colonization strategies that led to a different set of institutions, and these institutional differences are found to be the main reason for the income per capita differences between countries. Rodrik et al. (2004) extended Acemoglu et al. (2001) sample and also found that the quality of the institutions constitutes the primary reason for the cross-country income per capita differences. Bosker and Garretsen (2009) found that better institutions lead to higher long-term income per capita even after controlling for the effect of the neighboring institutions. In a recent application, Barro (2015) found that countries with “better rule of law” have higher growth rates suggesting that the econometric problems posed by country fixed effects may not be serious in samples within a long-time frame, something that contradicts the findings of Acemoglu et al. (2008, 2009) and is consistent with the modernization theory of Lipset (1959).^3^

It has been found that better institutions not only lead to long-term economic development but also aid is more effective in countries with good policies. The Monterrey consensus suggested that countries with sound institutions make more effective use of foreign aid (World Bank, 2003). In a seminal paper, Collier and Dollar (2002) found that the effectiveness of foreign aid depends on the quality of policies and institutions, whereas Kosack (2003) found that foreign aid is effective in improving the quality of life in countries with more democratic institutions, suggesting that aid allocation should be combined with the democratization effort of a given country (see also Burnside & Dollar, 2004; Dollar & Levin, 2006; Chong et al., 2009; Tebaldi & Mohan, 2010; Roodman, 2012). Overall, it has been found that aid empowers the poor in a good institutional setting and improves the functionings and capabilities of individuals.^4^

The main argument is that countries with better governance have better economic and social conditions, which then increase the capability and well-being of the individuals living in these countries. Therefore, the inclusion of the governance dimension to the already existing component list of HDI would be an important step. However, given that there are many proxies of governance (or institutional quality), the question then arises of which one of these proxies would be suitable for inclusion? Abu-Ismail et al. (2016) considers inclusion of the governance indicators to the HDI, however, they use voice and accountability and rule of law indicators from the World Governance Indicators, which are considered to be relative measures and do not capture over-time improvements or deteriorations in governance (Arndt & Oman, 2006; Knack, 2007) and they also do not allow any weight variation across dimensions used. In this paper, we examine a list of different possible governance proxies’ inclusion to the HDI components, with the use of SD spanning tests to examine whether their inclusion to the component list leads to welfare gains or losses.

## Stochastic spanning

SD is traditionally applied for comparing a pair of two distributions of given characteristics, and SD efficiency is a direct extension of SD to the case where full diversification is allowed. This is a multivariate problem of testing whether a given combination of characteristics (an index) is stochastically efficient relative to all mixtures of a discrete set of alternative characteristics (alternative indices). Pinar et al. (2013) use this methodology to test for the SD efficiency of the official HDI (the given index) concerning all possible combinations of weighting schemes (the set of indices) constructed from the set of components. Stochastic spanning is a generalization of SD efficiency because it involves the comparison of two sets of alternatives, while SD efficiency is a special case where one of the two choice sets is a singleton. We adopt stochastic spanning to test whether the inclusion of governance proxies to the original HDI components may lead to welfare gains or losses. If we were to add one such governance proxy as an additional component and fail to reject the null of spanning, then this additional component would not lead to additional welfare gains or losses. However, if we reject spanning, then there are combinations of the augmented index that includes all four components that dominate (or are dominated by) any combination of the components of the original HDI (i.e., income, health, and education). In that case, the inclusion of this governance indicator leads to welfare gains or losses. Gains will occur if the additional governance dimension covers an area where, on average, counties in the sample that experience gains would have had made considerable strides beyond those covered by the original HDI dimensions. The opposite would occur for losses as if the extra dimension would cover an area where, on average, the countries that experience losses would have fallen behind. To summarize, the idea behind stochastic spanning is that enlarging the set of potential outcomes would not lead to welfare gains or losses. If there are gains or losses, then spanning is rejected.

To provide some examples on how SD spanning may be useful, we offer a hypothetical scenario, where the application of the SD spanning methodology would be beneficial in this setting. Politicians usually highlight the social and economic progress in their electoral terms to increase their re-election chances. Therefore, governing politicians may refer to policy-specific wellbeing indices to highlight the progress made during their term. However, if the existing composite wellbeing index is not favourable, then they may decide to include new dimensions of wellbeing that would lead to a distribution of the new index that is larger than and dominates the distribution of every alternative. Even when the intention of the policymakers is not related to the above hypothetical case, the use of the SD spanning methodology enables one to identify such scenarios. Henceforth, policymakers would be aware of the potential implications of augmenting the existing list of indicators. In short, the notion of the SD spanning is suitable to check whether the inclusion of an indicator to the existing list of indicators leads to a distribution of a new index that is larger (smaller) than the distribution of every alternative, highlighting the fact that the inclusion of the new indicator may lead to distribution welfare gains (losses). Henceforth, SD spanning analyses could highlight indicators that may inflate/deflate cross-unit index outcomes and disallow misuse of index augmentation. On the other hand, if the inclusion of new indicators does not lead to a distribution of a new index that is larger (smaller) compared to that of every alternative may, it may be more suitable for inclusion to the existing composite index as its inclusion would not systematically inflate/deflate cross-unit index outcomes.

Below we will provide a formal presentation of stochastic spanning tests that are for testing for distributional welfare gains only as the spanning tests for distributional welfare losses could be easily obtained by reversing the order of two cumulative distributions that are used in the comparison. In the Appendix, we present the testing and computational framework that underlies our approach.

The welfare universe consists of *M* components with outcomes $X:=({x}_{1},\ldots, {x}_{M})$ with support bounded by ${X}^{M}:=[\underline{x},\overline{x}]$ where $-\infty <\underline{x}<\overline{x}<+\infty$. *X* can be chosen if it is a superset of the maximal support of the base components. The data correspond to observed values of the *M* different constituent components of well-being (or welfare). The components are treated as random variables with a discrete, state-dependent, joint probability distribution characterized by *R* mutually exclusive and exhaustive scenarios with probabilities ${p}_{r}>0, r=1,\dots ,R$. The feasible combinations of components are represented by a bounded polyhedral set, *M*-simplex $\Lambda :=\{\lambda \in {\mathbb{R}}_{+}^{M}:{1}_{M}^{T}\lambda =1\}$.

Let $F:{R}^{M}\to \left[\mathrm{0,1}\right]$ denote the continuous joint cumulative distribution functions (cdf’s) of *X* and $F\left(y,\lambda \right):=\int 1({X}^{T}\lambda \le y)dF(X)$ the marginal cdf for a combination $\lambda \in\Lambda$ where 1 is the indicator function. To define SD spanning and efficiency, we use the following integrated cdf:

1 $${\mathbf{F}}^{\left(2\right)}\left(\mathbf{x},\uplambda \right):={\int }_{-\infty }^{x}\mathbf{F}\left(\mathbf{y},\uplambda \right)\mathbf{d}\mathbf{y}={\int }_{-\infty }^{x}\left(\mathbf{x}-\mathbf{y}\right)\mathbf{d}\mathbf{F}(\mathbf{y},\uplambda )$$

This measure corresponds to first-order lower-partial moment of Bawa (1975), or expected shortfall, for a given threshold$x\in X$.^5^

This study focuses on the effects of changing the set of benchmark components of the HDI (i.e., income, health, and education). For this purpose, we introduce a non-empty polyhedral subset $K\subset\Lambda$. A polyhedral structure is analytically convenient and arises if we remove some of the base components or tighten the) linear constraints which define *L*.

### Stochastic spanning

The set of components $\Lambda$ is second-order stochastically spanned by subset $\mathrm{\rm K}\subset\Lambda$ if all combinations $\lambda \in\Lambda$ are weakly second-order stochastically dominated by some combinations of components $\kappa \in \mathrm{\rm K}$:

2 $$ \begin{gathered} \left( {\kappa { \succcurlyeq }_{F} \lambda \kappa \in K} \right):\forall \lambda \in \Lambda \Leftrightarrow \hfill \\ \left( {\left( {G\left( {x,\kappa ,\lambda ;F} \right) \le 0:\forall x \in {\mathcal{X}}} \right):\kappa \in K} \right):\forall \lambda \in \Lambda \hfill \\ \end{gathered} $$

3 $$G\left(x,\kappa ,\lambda ;F\right):={F}^{2}\left(x,\kappa \right)-{F}^{2}\left(x,\lambda \right)$$

We will use $R\left(\Lambda \right):=\left\{\mathrm{\rm K}\subseteq\Lambda :{\kappa \succcurlyeq }_{F}\lambda \kappa \in \mathrm{\rm K}\right):\forall\uplambda \in\Lambda \}$ to denote all relevant subsets that span $\Lambda$. Spanning occurs if and only if $\mathrm{\rm K}\in R\left(\Lambda \right)$. $R\left(\Lambda \right)$ is non-empty because it includes at least $\Lambda$; a span $\mathrm{\rm K}\in R\left(\Lambda \right)$ may itself be spanned by another span ${\mathrm{\rm K}}^{^{\prime}}\in R\left(\mathrm{\rm K}\right)\subseteq R\left(\Lambda \right)$.

Stochastic spanning occurs if the enlargement ($\Lambda -\mathrm{\rm K}$) does not change the efficient set (i.e., the most optimistic combination of the sub-components of the HDI, see Pinar et al., 2013, 2017 for the details), that is:

4 $$ {\text{\rm K}} \in R\left( {\Lambda } \right) \Leftarrow E\left( {\Lambda } \right) \subseteq {\text{\rm K}}. $$

The reverse relation generally does not hold, because the weak dominance relation does not possess the antisymmetric property. In other words, $E(\Lambda )$ always spans $\Lambda$, but it may be reducible by excluding equivalent elements. Consequently, $E(\Lambda )\subseteq \mathrm{\rm K}$ is a sufficient but not necessary condition for $\mathrm{\rm K}\in R\left(\Lambda \right)$. Also, the sufficient condition $E(\Lambda )\subseteq \mathrm{\rm K}$ is not practical because $E(\Lambda )$ is generally non-convex and disconnected, which makes it difficult to identify all its elements and test the sufficient condition directly. On the contrary, a small polyhedral span $\mathrm{\rm K}\in R\left(\Lambda \right)$ could be used as a practical approximation to the intractable efficient set $E(\Lambda )$.

We use the following scalar-valued functional of the population cdf as a measure for deviations from stochastic spanning:

5 $$\eta \left(F\right):=\underset{\lambda \in \Lambda }{\mathrm{sup}}\underset{\kappa \in K}{\mathrm{inf}}\underset{x\in \mathcal{X}}{\mathrm{sup}}G(x,\kappa ,\lambda ;F)$$

where $\lambda ,\kappa \ge {w}_{min}$ and the minimum weight (i.e., ${w}_{min}$) allocated to well-being dimensions could be set by decision-makers. The outer maximization searches for a feasible combination $\lambda \in \Lambda$ that is not weakly dominated by a combination $\kappa \in K$. If $\eta \left(F\right)=0$, then no such combination of components exists, and *K* spans $\Lambda$; if $\eta \left(F\right)>0$, then stochastic spanning does not occur. The formulation of the test statistic based on (5) and its implementation is given in the Appendix.

In the paper, we use cross-sectional instead of time-series data used by Arvanitis et al. (2019) or Linton et al. (2005). In Appendix A, we discuss the details of how the application of SD spanning to cross-sectional data differs from the large time-series case. An analogous with the Arvanitis et al. (2019) asymptotic theory could be developable under the assumption of (weak) exchangeability of the random variables involved.

## Empirical application

### Data

We will use the United Nations Development Program's HDI and its components—health, education, and income indices for 2010, 2011, 2012, 2013, 2014, and 2015.^6^ The HDI is obtained as the geometric average of the three sub-indices, where each index is obtained through a normalization procedure by setting minimum and maximum (goalposts) to set the values between 0 and 1:

$$\mathrm{Dimension\;index}=\frac{\mathrm{Actual\;value}-\mathrm{Minimum\;value}}{\mathrm{Maximum\;value}-\mathrm{Minimum\;value}}$$

The health sub-index is measured by life expectancy (LE) at birth, and the normalized sub-index outcomes are obtained by using minimum and maximum goalposts of 20 and 85 years, respectively. Hence, the health index (HI) outcome of a given country is obtained by using the following normalization procedure $\mathrm{HI}=\frac{\mathrm{LE}-20}{85-20}$ where LE is the life expectancy at birth for a given country.

The education sub-index is measured by the mean years of schooling (MYS) for adults aged 25 years and the expected years of schooling (EYS) for children of school entering age. The index values for MYS and EYS (MYSI and EYSI respectively) are obtained by using a minimum value of zero and maximum values of 15 and 18 years such as $\mathrm{MYSI}=\frac{\mathrm{MYS}-0}{15-0}$ and $\mathrm{EYSI}=\frac{\mathrm{EYS}-0}{18-0}$, respectively. Then, two indices are combined into an education index (EI) using the arithmetic mean, i.e., $\mathrm{EI}=\frac{\mathrm{MYSI}+\mathrm{EYSI}}{2}$.

The standard of living dimension is measured by gross national income per capita. The minimum and maximum goalposts for gross national income (GNI) per capita are $100 and $75,000, respectively. The income index (II) is then calculated using the normalization procedure $$\mathrm{II}=\frac{\mathrm{ln}\left(\mathrm{GNI per capita}\right)-\mathrm{ln}(100)}{\mathrm{ln}\left(\mathrm{75,000}\right)-\mathrm{ln}(100)}$$.

To test for inclusion in the set of HDI dimensions, we use twelve proxies for governance (institutional quality) that are extensively used in the empirical literature. In particular, we use the corruption perceptions index (CPI) from Transparency International; the democracy index from the Polity IV database (which is a combined index of institutionalized democracy and autocracy indices); property rights from the Heritage Foundation (HF hereafter) database (Miller & Kim, 2016); the overall economic freedom index, judicial independence, protection of property rights, legal system and property rights, extra payments/bribes/favoritism (bribes hereafter), and regulation components from the Economic Freedom of the World (EFW) database of the Fraser Institute (FI hereafter) (Gwartney et al., 2016) and the property rights, civil liberties, and their combined overall score from the Freedom House (FH) database. Table 1 lists the details of these proxies and each proxy's range. Indicators with a high score imply better governance in a given country (i.e., better property rights, higher judicial independence, better democracy, less corruption, and so on). We standardize each of the institutional proxies to range between 0 and 1 (similar to that of HDI components) with a higher score representing a better institutional quality outcome.^7^

**Table 1:** List of governance indicators

Governance measure	Source	Range	Available via:
Corruption perceptions index	Transparency International	0–100	https://www.transparency.org/cpi
Democracy index	Polity IV	− 10 to + 10	http://www.systemicpeace.org/inscrdata.html
Property rights	The Heritage Foundation	0–10	Miller and Kim (2016) http://www.heritage.org/index/
Economic Freedom	Fraser Institute	0–10	Gwartney et al. (2016) https://www.fraserinstitute.org/economic-freedom/dataset
Judicial independence	Fraser Institute	0–10	Gwartney et al. (2016) https://www.fraserinstitute.org/economic-freedom/dataset
Protection of property rights	Fraser Institute	0–10	Gwartney et al. (2016) https://www.fraserinstitute.org/economic-freedom/dataset
Legal System & Property Rights	Fraser Institute	0–10	Gwartney et al. (2016) https://www.fraserinstitute.org/economic-freedom/dataset
Extra payments/bribes/favouritism	Fraser Institute	0–10	Gwartney et al. (2016) https://www.fraserinstitute.org/economic-freedom/dataset
Regulation	Fraser Institute	0–10	Gwartney et al. (2016) https://www.fraserinstitute.org/economic-freedom/dataset
Property rights	Freedom house	0–40	https://freedomhouse.org/report/freedom-world/freedom-world-2017
Civil liberties	Freedom house	0–60	https://freedomhouse.org/report/freedom-world/freedom-world-2017
Overall score (Property rights + Civil liberties)	Freedom house	0–100	https://freedomhouse.org/report/freedom-world/freedom-world-2017

Table 2 presents the descriptive statistics for all the normalized sub-components for 2015. Among the sub-components of the HDI, the health index has the highest mean (median) followed by the income and education indices. However, the average achievements in institutional quality components show variation across proxies. For instance, we have three sub-components that measure the enforcement and protection of property rights (i.e., property rights components from the HF, FI, and FH). The average level of protection of property rights with the FH proxy is relatively higher compared to the other proxies from the HF and FI. Also, the former proxy displays negative skewness, whereas the other two proxies display positive skewness suggesting that the distributions of these proxies are fairly asymmetric. Another interesting feature of the descriptive statistics is that the original components of the HDI always display negative skewness, whereas some of the institutional quality proxies display negative, and some others display positive skewness. These features of the data sets justify our model-free approach that uses information beyond the second moment of the distributions in question.

**Table 2:** Summary statistics

	Mean	Median	Standard deviation	Skewness	Obs
Health index	0.790	0.822	0.128	− 0.668	188
Education index	0.639	0.659	0.174	− 0.344	188
Income index	0.687	0.702	0.180	− 0.289	188
CPI	0.430	0.370	0.201	0.790	162
Polity IV—Democracy index	0.718	0.850	0.307	− 1.008	156
HF—Property rights	0.422	0.350	0.250	0.654	177
FI—Economic Freedom	0.679	0.686	0.091	− 0.675	158
FI—Judicial independence	0.501	0.475	0.211	0.312	151
FI—Property rights	0.560	0.536	0.166	0.281	151
FI—Legal system & Property rights	0.525	0.508	0.156	0.415	158
FI—Extra payments/bribes/favouritism	0.445	0.411	0.177	0.782	150
FI—Regulation	0.699	0.703	0.108	− 0.689	158
FH—Property rights	0.601	0.675	0.320	− 0.392	188
FH—Civil liberties	0.604	0.617	0.274	− 0.223	188
FH—Property rights & Civil liberties	0.603	0.635	0.290	− 0.292	188

Table 3 presents the correlation coefficients between the sub-components of HDI (i.e., health, education, and income indices) with the other institutional quality proxies in 2015. It has been argued that if the dimensions are highly and positively correlated, any index constructed by using these dimensions would be redundant (see e.g., McGillivray, 2005; Cahill, 2005; Bérenger and Verdier-Chouchane, 2007; Foster et al., 2013 among many others that examine the redundancy of the HDI by using correlation analysis). Since the correlation coefficients between the sub-components of the original HDI and the other institutional quality proxies are relatively low compared with the correlation coefficients among the members of the original set of HDI dimensions and also given the variation exhibited by the distributions of the institutional quality proxies (i.e., differences in mean, standard deviation and skewness measures across governance indicators), we can expect that the inclusion of an additional institutional quality proxy to the original set of sub-components of HDI may lead to welfare gains or losses beyond that provided by the sub-components of the HDI.

**Table 3:** Correlation coefficients between governance proxies and sub-components of the HDI

	Health	Education	Income	Obs
Health	1.000***	0.806***	0.795***	188
Education	0.806***	1.000***	0.839***	188
Income	0.795***	0.839***	1.000***	188
CPI	0.674***	0.705***	0.717***	162
Polity IV—Democracy	0.300***	0.333***	0.179**	156
HF—Property rights	0.624***	0.636***	0.662***	177
FI—Economic Freedom	0.580***	0.593***	0.559***	158
FI—Judicial	0.486***	0.511***	0.570***	151
FI—Property rights	0.528***	0.539***	0.599***	151
FI—Legal system & Property rights	0.654***	0.713***	0.691***	158
FI—Extra payments/bribes/favouritism	0.565***	0.537***	0.604***	150
FI—Regulation	0.423***	0.506***	0.506***	158
FH—Property rights	0.487***	0.526***	0.395***	188
FH—Civil liberties	0.536***	0.593***	0.469***	188
FH—Property rights & Civil liberties	0.520***	0.569***	0.441***	188

### Results

In this subsection, we present our findings on testing the null hypothesis of SD spanning, namely that the inclusion of additional institutional quality proxies to the existing set of HDI components does not result in a welfare improvement or deterioration. To avoid corner solutions, we set two minimum weights that well-being dimensions could take while obtaining SD efficient and spanning weights (i.e., 0.1 and 0.15) in all the applications.

We proceed by first finding the SD efficient weights for the components of the benchmark HDI, health, education, and income (i.e., the implicit weights that lead to the most optimistic and pessimistic welfare measurement). For all the years, we find that the health and education dimensions receive relatively a higher weight in the optimistic and pessimistic scenario, respectively (see Pinar et al., 2017, for further discussion on this). Once we obtain the SD efficiency weights, we test whether the inclusion of the governance proxies, one by one, leads to SD spanning or welfare gains or losses with the inclusion of the indicator. Table 4 summarizes the results obtained SD spanning test results with the inclusion of twelve governance indicators. The detailed set of results for each indicator is given in Tables S1-S12 in Supplementary Materials A. Panels A and B of Table 4 provide the spanning results for the dominating and dominated scenarios when spanning is rejected suggesting that the inclusion of a governance indicator leads to welfare gains or losses with some admissible weights, respectively. There are three general outcomes obtained with these applications: (1) ‘fail to reject spanning’ in the dominating scenario but ‘reject spanning’ in the dominated scenario, (2) ‘reject spanning’ in the dominating scenario but ‘fail to reject spanning’ in the dominated scenario, and (3) ‘fail to reject spanning’ in both scenarios. The first and second cases suggest that combinations of governance indicators with three components of the HDI with some admissible weights lead to welfare losses and gains, respectively. The third case suggests that a combination of governance indicators with the other three components of the HDI does not lead to welfare gains or losses irrespective of any admissible weights used.

**Table 4:** Stochastic spanning test results with the inclusion of governance indicators to the HDI

	CPI	Polity IV	HF-PR	FI-Econ	FI-Judicial	FI-PR	FI-LSPR	FI-Bribes	FI-Reg	FH-PR	FH-CL	FH-PRCL
*Panel A: Spanning test results for the second-order stochastically dominating scenario with the inclusion of an indicator*												
2015	S	RS	S	RS	S	S	S	S	RS	S	S	S
2014	S	RS	S	RS	S	S	S	S	RS	S	S	S
2013	S	RS	S	RS	S	S	S	S	RS	S	S	S
2012	S	RS	S	RS	S	S	S	S	RS	S	S	S
2011	S	RS	S	RS	S	S	S	S	RS	S	S	S
2010	S	RS	S	RS	S	S	S	S	RS	S	S	S
*Panel B: Spanning test results for the second-order stochastically dominated scenario with the inclusion of an indicator*												
2015	RS	S	RS	S	RS	RS	RS	RS	S	RS	RS	RS
2014	RS	S	RS	S	RS	RS	RS	RS	S	RS	RS	RS
2013	RS	S	RS	S	RS	RS	RS	RS	S	RS	RS	RS
2012	RS	S	RS	S	RS	RS	RS	RS	S	RS	S	RS
2011	RS	S	RS	S	RS	RS	RS	RS	S	S	S	S
2010	RS	S	RS	S	RS	RS	RS	RS	S	S	S	S

S: Fail to reject spanning at the 5% level. RS: Reject spanning at the 5% level

CPI: Corruption Perceptions Index; Polity IV: Democracy index from Polity IV; HF-PR: property rights from the Heritage Foundation; FI-Econ: overall economic freedom index from the Economic Freedom of the World database of the Fraser Institute; FI: Judicial: Judicial independence index from the Economic Freedom of the World database of the Fraser Institute; FI-PR: Protection of property rights index from the Economic Freedom of the World database of the Fraser Institute; FI-LSPR: Legal system and property rights from the Economic Freedom of the World database of the Fraser Institute; FI-Bribes: Extra payments/bribes/favouritism index from the Economic Freedom of the World database of the Fraser Institute; FI-Regulation: Regulation index from the Economic Freedom of the World database of the Fraser Institute; FH-PR: Property rights index from the Freedom House; FH-CL: Civil liberties index from the Freedom House; FH-PRCL: Property rights and civil liberties index from the Freedom House

The results differ depending on the type of institutional quality proxy used. In particular, we find that for six of the twelve institutional quality proxies (i.e., CPI, property rights index from HF, judicial independence, property rights, legal system and property rights and bribery components from the FI), we fail to reject spanning for the dominating scenario and reject spanning with the dominated scenario for all years. In other words, a combination of these governance indicators with the three components of HDI leads to distributional welfare losses. On the other hand, with other three governance indicators (i.e., the Polity IV democracy index, economic freedom and regulation indices from the FI), we reject the null hypothesis of spanning for the dominating scenario and fail to reject the null hypothesis of spanning for the dominated scenario suggesting that a combination of these proxies with the three components of HDI will lead to distributional welfare gains. Finally, with the civil liberties, property rights, and combination of civil liberties and property rights variables from FH, we fail to reject spanning in both cases when data from 2010 and 2011 (and 2012 for civil liberties indicator) are used, suggesting that a combination of governance indicators with the other three components of HDI does not lead to welfare gains or losses irrespective of any admissible weights used. When data from 2013, 2014, and 2015 are used for the latter three governance indicators, we find that their combination with the components of the original HDI leads to distributional welfare losses with some admissible weights. Overall, the results suggest that there exists a combination of the four components (i.e., the three components of HDI and an additional governance component), where the distribution of the augmented HDI scores second-order stochastically dominates (dominated by) the distribution of the HDI without the governance proxy when three (six) governance proxies are used in all years with some admissible weights. The combinations of components for the cases where spanning with the governance indicators is rejected between 2010 and 2015 when minimum weights for dimensions are set to 0.1 and 0.15 are given in Tables S20-S23 in Supplementary Materials B.

**Fig. 1:**
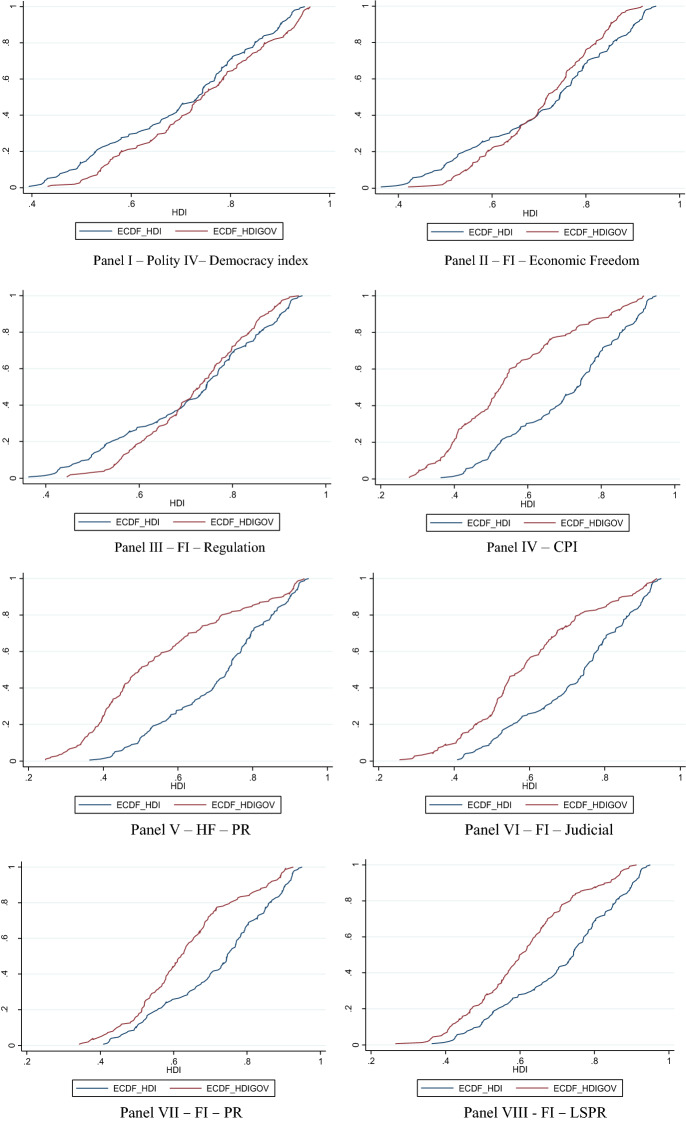

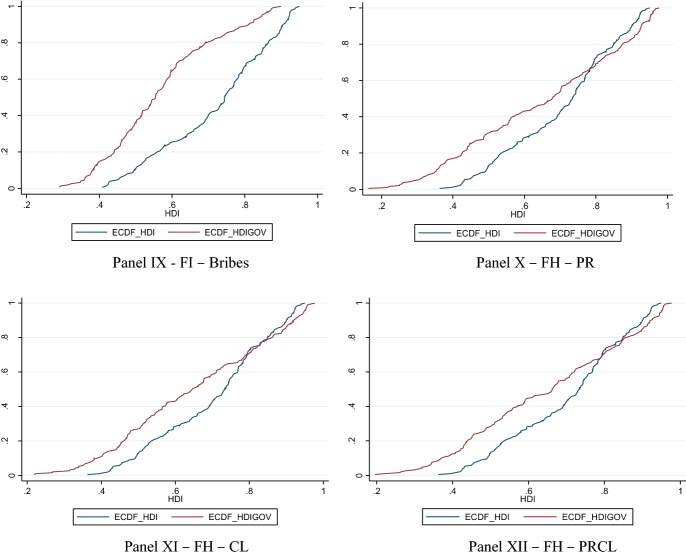
Empirical cumulative distribution of the benchmark HDI and HDI scores with governance indicators when lower bound weight is set to 0.15 (ECDF_HDI and ECDF_HDIGOV, respectively)

To show the distributional welfare gains and losses with the combinations of governance indicators with the three components of HDI, the twelve panels (I-XII) in Fig. 1 graph the empirical distributions of the equally-weighted HDI scores in 2015 without the governance indicator, presented as ECDF_HDI, and the empirical distributions of the composite index scores with the proposed weights in Appendix Table B2 and B4 in 2015 (which are presented as ECDF_HDIGOV), respectively. In the first three panels (I-III) of Fig. 1, the welfare distribution of the augmented index second-order stochastically dominates the benchmark HDI when the democracy index of Polity IV, economic freedom, and regulation indices from FI are included as a governance proxy with some admissible weights. However, when the corruption perceptions index, property rights from HF, judicial independence, property rights, legal system and property rights, bribes indices from FI are used as governance proxies, the augmented index is dominated by the benchmark HDI at the first-order (see panels IV-IX of Fig. 1). Finally, the inclusion of proxies from HF (property rights, civil liberties, and combined property rights and civil liberties indices) makes the augmented index to be second-order stochastically dominated by the benchmark HDI (see panels X–XII of Fig. 1). The empirical distribution of the benchmark HDI lies below the empirical distributions of the composite indices obtained with governance proxies from HF at the initial scores but cuts the empirical distributions of the augmented indices from below at the higher composite index values suggesting second-order dominance of benchmark HDI over the augmented indices. In sum, the inclusion of proxies that are seemingly measuring the same concept may lead to distinctively different distributions of welfare when these proxies are combined with the HDI components. That augmentation that leads to gains would suggest that most countries would have higher achievements in this extra dimension when compared with the original benchmark combination, and the opposite would be the case for losses.

Beyond the distinctive welfare distributions with the inclusion of governance indicators, we also examine whether the inclusion of governance indicators lead to distinctive rankings differences when compared with the country rankings obtained with the official HDI. To do this, we provide analysis of country rankings when the augmented indices and the benchmark HDI index are used to rank countries in 2015. In particular, when we make the comparisons, we use the composite index outcomes obtained with the equally-weighted HDI and the index outcomes obtained with the weights reported in Tables B2 and B4 for the year 2015. Even though the rankings obtained with the augmented indices and the official HDI are highly correlated since the governance indicators are positively correlated with the sub-components of the HDI (see Table 3),^8^ the distributions of augmented composite indices are distinctively different from the benchmark HDI (see Fig. 1). Furthermore, there exist major rank reversals between the augmented composite indices and the benchmark HDI. Table 5 provides the 20 countries that would experience the biggest and lowest average upward and downward changes with the augmented indices that lead to welfare gains and losses relative to their position based on the benchmark HDI in 2015 when the minimum weight is set to 0.15.^9^ The major rank reversals that we observe when governance components are included suggest that there are major differences in governance achievements across countries. For instance, Nicaragua (Saudi Arabia) would have moved 22 positions upward (33 positions downward), on average, with the augmented composite indices obtained with the inclusion of three governance proxies (i.e., three governance proxies whose inclusion leads to welfare gains with some admissible weights) compared to the benchmark equally-weighted HDI. Similarly, on average, Cape Verde (Russia) would have moved 38 positions upward (40 positions downward) with the augmented indices compared to the benchmark indices when nine governance proxies whose inclusion leads to major distributional welfare losses.^10^

**Table 5:** Countries that experienced the largest upward and downward movements in their rankings with the augmented indices compared to the HDI rankings

Average ranking change with the augmented indices that lead welfare gain				Average ranking change with the augmented indices that lead welfare loss			
Country	Upward Δ	Country	Downward Δ	Country	Upward Δ	Country	Downward Δ
Nicaragua	22	Saudi Arabia	33	Cape Verde	38	Russia	40
Jamaica	21	Iran	32	Bhutan	36	Iran	35
Peru	19	Argentina	23	South Africa	36	Saudi Arabia	30
Honduras	18	Russia	22	Namibia	35	Argentina	28
Guatemala	16	Algeria	21	Rwanda	34	Kazakhstan	26
Macedonia	15	Oman	19	Botswana	33	Lebanon	25
Albania	14	Kuwait	19	Ghana	30	Azerbaijan	21
Philippines	13	Egypt	17	India	30	Singapore	21
Cabo Verde	13	Brazil	17	Lesotho	27	Ukraine	19
Bhutan	13	Azerbaijan	16	Senegal	25	Vietnam	18
Dominican Rep	13	Kazakhstan	16	Benin	20	Bahrain	18
Mauritius	12	Gabon	15	Uruguay	18	Algeria	18
Georgia	12	Ukraine	14	Liberia	18	Ecuador	18
Armenia	11	Qatar	13	Malawi	18	UAE	18
Rwanda	11	UAE	12	Tunisia	17	Kuwait	18
Mongolia	11	Greece	11	Jamaica	16	Qatar	17
El Salvador	11	Bahrain	10	Zambia	16	Greece	15
Jordan	9	Trinidad & Tobago	9	Mauritius	16	Korea, Rep	15
Colombia	9	South Africa	8	Tanzania	14	Italy	15
Mexico	9	Cameroon	8	Syria	14	Hungary	14

At first glance, since the correlation coefficients between the augmented indices and the equally-weighted HDI are high and positive, one might have suspected that the inclusion of these governance indicators may have been redundant based on the redundancy literature (see e.g., McGillivray, 2005; Foster et al., 2013). However, when we examined the composite achievement levels with the inclusion of governance indicators, we find that combinations of governance indicators with the components of the original HDI result in distributional gains and losses compared to the benchmark HDI. Furthermore, when we compare the rankings obtained with the composite indices that include governance indicators with the equally-weighted HDI rankings, we also observe major rank reversals for most developing countries. This suggests that the inclusion of some of the governance indicators not only leads to a marked increase (decrease) in welfare outcomes but also provides additional variation in the country ranking analysis.

### Robustness analysis

In the baseline analysis, we imposed a lower bound weight to test for stochastic spanning. In particular, we imposed lower bound weights of 0.10 and 0.15 to test for spanning, which then imposed respective upper bound weights of 0.7 and 0.55, respectively. In this section, we carry out an additional set of robustness analyses. Firstly, we set the lower bound weight to 0.2 so that the weight variation between the four dimensions ranges between 0.2 and 0.4. The results are presented in Table 6. Even though we imposed a higher lower bound weight, our results remain the same with respect to the cases when lower bound weights were set to 0.1 and 0.15 with few exceptions. The exceptions are as follows. The spanning tests are rejected for 2010 and 2012 when civil liberties index from the Freedom House (FH-CL) was included, rejected for 2011 when the property rights index from the Freedom House (PH-CL) was included, and rejected for 2012 when property rights and civil liberties index from the Freedom House (FH-PRCL) was included (see Table S13 for the detailed set of results for each indicator in Supplementary Materials A). The combinations of components for the cases where spanning with the governance indicators is rejected between 2010 and 2015 when minimum weight for dimensions is set to 0.2, are given in Tables S24 and S25 in Supplementary Materials B.

**Table 6:** Stochastic spanning test results with the inclusion of governance indicators to the HDI when lower bound weight is 0.2

	CPI	Polity IV	HF-PR	FI-Econ	FI-Judicial	FI-PR	FI-LSPR	FI-Bribes	FI- Reg	FH-PR	FH-CL	FH-PRCL
*Panel A: Spanning test results for the second-order stochastically dominating scenario with the inclusion of an indicator*												
2015	S	RS	S	RS	S	S	S	S	RS	S	S	S
2014	S	RS	S	RS	S	S	S	S	RS	S	S	S
2013	S	RS	S	RS	S	S	S	S	RS	S	S	S
2012	S	RS	S	RS	S	S	S	S	RS	S	S	S
2011	S	RS	S	RS	S	S	S	S	RS	S	S	S
2010	S	RS	S	RS	S	S	S	S	RS	S	S	S
*Panel B: Spanning test results for the second-order stochastically dominated scenario with the inclusion of an indicator*												
2015	RS	S	RS	S	RS	RS	RS	RS	S	RS	RS	RS
2014	RS	S	RS	S	RS	RS	RS	RS	S	RS	RS	RS
2013	RS	S	RS	S	RS	RS	RS	RS	S	RS	RS	RS
2012	RS	S	RS	S	RS	RS	RS	RS	S	RS	RS	RS
2011	RS	S	RS	S	RS	RS	RS	RS	S	RS	S	S
2010	RS	S	RS	S	RS	RS	RS	RS	S	S	RS	RS

S: Fail to reject spanning at the 5% level. RS: Reject spanning at the 5% level

CPI: Corruption Perceptions Index; Polity IV: Democracy index from Polity IV; HF-PR: property rights from the Heritage Foundation; FI-Econ: overall economic freedom index from the Economic Freedom of the World database of the Fraser Institute; FI: Judicial: Judicial independence index from the Economic Freedom of the World database of the Fraser Institute; FI-PR: Protection of property rights index from the Economic Freedom of the World database of the Fraser Institute; FI-LSPR: Legal system and property rights from the Economic Freedom of the World database of the Fraser Institute; FI-Bribes: Extra payments/bribes/favouritism index from the Economic Freedom of the World database of the Fraser Institute; FI-Regulation: Regulation index from the Economic Freedom of the World database of the Fraser Institute; FH-PR: Property rights index from the Freedom House; FH-CL: Civil liberties index from the Freedom House; FH-PRCL: Property rights and civil liberties index from the Freedom House

While testing for stochastic spanning, we have not imposed any weight constraints while obtaining the SDE weights (i.e., SDE weights obtained for the three original HDI components before the spanning test). We carry out the second set of robustness analysis when we impose lower bound weights of 0.2 and 0.25 to the original HDI dimensions to obtain SDE weights and then test for stochastic spanning when we impose lower bound weights of 0.1, 0.15 and 0.2 for the four components (i.e., three original components and one governance indicator). Table 7 reports the results when we impose lower bound weights of 0.25 to the original HDI dimensions while obtaining SDE weights. Part I, II and III reports the results when the whole set of components have imposed lower bound weights of 0.2, 0.15 and 0.1, respectively. The results in Part I of Table 7 are similar to those reported in Table 6 when no lower bound weight were imposed on the original HDI components while obtaining SDE weights. Similarly, the results obtained in parts II and III of Table 7 are similar to those reported in Table 4 when no lower bound weight is imposed on the original HDI components while obtaining SDE weights. We do not report the spanning test results obtained for the case when we impose lower bound weights of 0.2 to the original HDI dimensions while obtaining SDE weights as the findings are the same as the ones reported in Table 7. Test statistics for each scenario are reported in Tables S14-S19 in Supplementary Materials A. The weights for the spanning scenarios are not reported as the weights are similar to those reported in Tables S20-S25 in Supplementary Materials B (see Tables S20 and S22 when lower bound weight is set to 0.10; Tables S21 and S23 when lower bound weight is set to 0.15; and Tables S24 and S25 when lower bound weight is set to 0.20).

**Table 7:** Stochastic spanning test results with the inclusion of governance indicators to the HDI when SDE weights obtained with a lower bound weight of 0.25

	CPI	Polity IV	HF-PR	FI-Econ	FI-Judicial	FI-PR	FI-LSPR	FI-Bribes	FI- Reg	FH-PR	FH-CL	FH-PRCL
**Part I. Spanning results when lower bound weights for the whole components are 0.2**												
*Panel A: Spanning test results for the second-order stochastically dominating scenario with the inclusion of an indicator*												
2015	S	RS	S	RS	S	S	S	S	RS	S	S	S
2014	S	RS	S	RS	S	S	S	S	RS	S	S	S
2013	S	RS	S	RS	S	S	S	S	RS	S	S	S
2012	S	RS	S	RS	S	S	S	S	RS	S	S	S
2011	S	RS	S	RS	S	S	S	S	RS	S	S	S
2010	S	RS	S	RS	S	S	S	S	RS	S	S	S
*Panel B: Spanning test results for the second-order stochastically dominated scenario with the inclusion of an indicator*												
2015	RS	S	RS	S	RS	RS	RS	RS	S	RS	RS	RS
2014	RS	S	RS	S	RS	RS	RS	RS	S	RS	RS	RS
2013	RS	S	RS	S	RS	RS	RS	RS	S	RS	RS	RS
2012	RS	S	RS	S	RS	RS	RS	RS	S	RS	RS	RS
2011	RS	S	RS	S	RS	RS	RS	RS	S	RS	S	S
2010	RS	S	RS	S	RS	RS	RS	RS	S	S	RS	RS
**Part II. Spanning results when lower bound weights for the whole components are 0.15**												
*Panel A: Spanning test results for the second-order stochastically dominating scenario with the inclusion of an indicator*												
2015	S	RS	S	RS	S	S	S	S	RS	S	S	S
2014	S	RS	S	RS	S	S	S	S	RS	S	S	S
2013	S	RS	S	RS	S	S	S	S	RS	S	S	S
2012	S	RS	S	RS	S	S	S	S	RS	S	S	S
2011	S	RS	S	RS	S	S	S	S	RS	S	S	S
2010	S	RS	S	RS	S	S	S	S	RS	S	S	S
*Panel B: Spanning test results for the second-order stochastically dominated scenario with the inclusion of an indicator*												
2015	RS	S	RS	S	RS	RS	RS	RS	S	RS	RS	RS
2014	RS	S	RS	S	RS	RS	RS	RS	S	RS	RS	RS
2013	RS	S	RS	S	RS	RS	RS	RS	S	RS	RS	RS
2012	RS	S	RS	S	RS	RS	RS	RS	S	RS	S	RS
2011	RS	S	RS	S	RS	RS	RS	RS	S	S	S	S
2010	RS	S	RS	S	RS	RS	RS	RS	S	S	S	S
**Part III. Spanning results when lower bound weights for the whole components are 0.10**												
*Panel A: Spanning test results for the second-order stochastically dominating scenario with the inclusion of an indicator*												
2015	S	RS	S	RS	S	S	S	S	RS	S	S	S
2014	S	RS	S	RS	S	S	S	S	RS	S	S	S
2013	S	RS	S	RS	S	S	S	S	RS	S	S	S
2012	S	RS	S	RS	S	S	S	S	RS	S	S	S
2011	S	RS	S	RS	S	S	S	S	RS	S	S	S
2010	S	RS	S	RS	S	S	S	S	RS	S	S	S
*Panel B: Spanning test results for the second-order stochastically dominated scenario with the inclusion of an indicator*												
2015	RS	S	RS	S	RS	RS	RS	RS	S	RS	RS	RS
2014	RS	S	RS	S	RS	RS	RS	RS	S	RS	RS	RS
2013	RS	S	RS	S	RS	RS	RS	RS	S	RS	RS	RS
2012	RS	S	RS	S	RS	RS	RS	RS	S	RS	S	RS
2011	RS	S	RS	S	RS	RS	RS	RS	S	S	S	S
2010	RS	S	RS	S	RS	RS	RS	RS	S	S	S	S

S: Fail to reject spanning at the 5% level. RS: Reject spanning at the 5% level

CPI: Corruption Perceptions Index; Polity IV: Democracy index from Polity IV; HF-PR: property rights from the Heritage Foundation; FI-Econ: overall economic freedom index from the Economic Freedom of the World database of the Fraser Institute; FI: Judicial: Judicial independence index from the Economic Freedom of the World database of the Fraser Institute; FI-PR: Protection of property rights index from the Economic Freedom of the World database of the Fraser Institute; FI-LSPR: Legal system and property rights from the Economic Freedom of the World database of the Fraser Institute; FI-Bribes: Extra payments/bribes/favouritism index from the Economic Freedom of the World database of the Fraser Institute; FI-Regulation: Regulation index from the Economic Freedom of the World database of the Fraser Institute; FH-PR: Property rights index from the Freedom House; FH-CL: Civil liberties index from the Freedom House; FH-PRCL: Property rights and civil liberties index from the Freedom House

Overall, our findings are robust to the increased lower bound weights for the whole set of components with few exceptions (see Table 6). Furthermore, imposing a lower bound weight to the original components while obtaining the SDE weights before the spanning analysis yield the same results with imposing no SDE weight constraints (see Table 7).

## Concluding remarks

This paper applied SD spanning testing to examine the inclusion of additional indicators to the component list of the HDI. In particular, we tested for the inclusion of twelve governance indicators to the component list of the HDI, since governance is deemed to be a socially and economically important factor for development. The SD spanning tests allowed us to examine whether the inclusion of any governance indicator provides additional welfare gains or losses to the benchmark HDI index. We compared the empirical distribution of any combination of the three dimensions of the HDI with the empirical cumulative distribution of any combination of sub-components of the HDI and governance indicator. We find that choice of the governance proxy to be included in the component list of the HDI matters significantly as the inclusion of some indicators to the component list leads to distributional welfare gains, and some others’ inclusion leads to distributional welfare losses. Our findings suggest that even though the inclusion of governance dimension to the HDI is worthwhile, policymakers should be cautious about the proxy that they may choose given the fact that the inclusion of different indicators that are seemingly measuring the same concept leads to distinctively different distributions of welfare.

In the paper, we only examined the inclusion of governance indicators to the list of components of the HDI. Other sets of important factors may also be tested for inclusion to the HDI, such as the set of environmental factors. We leave the SD spanning testing of such additional factors for future research.

### Electronic supplementary material

Below is the link to the electronic supplementary material.

Supplementary file1 (DOCX 175 KB)

## Appendix A: Statistical theory

We use the test statistic developed in Arvatinis et al. (2019). Let $q({\eta }_{\infty },1-\alpha )$ denote the $1-\alpha$ quantile of the distribution of ${\eta }_{\infty }$ for any significance level $\alpha \in ]\mathrm{0,1}[.$ The basic decision rule to reject ${\mathbf{H}}_{0}$ against ${\mathbf{H}}_{1}$ if and only if ${\eta }_{T}>q({\eta }_{\infty },1-\alpha )$ is infeasible due to the dependence of $q({\eta }_{\infty },1-\alpha )$ on the latent cdf of *F*. However, feasible decision rules can be obtained by using a subsampling procedure to estimate $q({\eta }_{\infty },1-\alpha )$ from the data.

To implement the subsampling procedure, we begin by generating ($$N-{b}_{N}+1)$$ maximally overlapping subsamples of $${b}_{1}\in {\mathbb{N}}_{1}$$ consecutive observations, $${s}_{{b}_{N};N,n}:={({X}_{s})}_{s=n}^{n+{b}_{N}-1}$$, $$n=1,\dots ,N-{b}_{N}+1$$, and compute test scores $${\eta }_{{b}_{N};N,n}=\sqrt{{b}_{N}}\eta ({F}_{{b}_{N};N,n})$$ for each subsample, where $${F}_{{b}_{N};N,n}$$ denotes the empirical joint cdf constructed from $${s}_{{b}_{N};N,n}$$, $$n=1,\dots ,N-{b}_{N}+1$$. The distribution of subsample test scores can be described by the following cdf and quantile function:

6 $${S}_{N,{b}_{N}}\left(y\right):=\frac{1}{N-{b}_{N}+1}\sum_{n=1}^{N-{b}_{N}+1}1\left({\eta }_{{b}_{N};N,n}\le y\right);$$

7 $${q}_{N,{b}_{N}}\left(1-\alpha \right):=\underset{y}{\mathrm{inf}} \left\{y:{S}_{N,{b}_{N}}\left(y\right)\ge 1-\alpha \right\}.$$

Our decision rule is to reject the null ${\mathbf{H}}_{0}:\eta \left(F\right)=0$ against the alternative ${\mathbf{H}}_{1}:\eta \left(F\right)>0$ at a significance level of $\alpha \in ]\mathrm{0,1}[$ if and only if${\eta }_{N}>{q}_{N,{b}_{N}}(1-\alpha )$, or, equivalently,$1-{S}_{N,{b}_{N}}\left({\eta }_{N}\right)<\alpha$. This subsampling routine is asymptotically exact and consistent under reasonable assumptions on the subsample length and significance level.

Although the test has the asymptotically correct size, simulation exercises show that the quantile estimates ${q}_{N,{b}_{N}}(1-\alpha )$ may be biased and sensitive to the subsample size ${b}_{N}$ in finite samples of realistic dimensions (*M*). To correct for small-sample bias and reduce the sensitivity to the choice of ${b}_{N}$, we propose a regression-based bias-correction method that is motivated by our observations from simulation exercises. For a given significance level α, we compute the quantiles ${q}_{N,{b}_{N}}(1-\alpha )$ for a reasonable range of the subsample size ${b}_{N}$. Next, we estimate the intercept and slope of the following regression line using ordinary least squares (OLS) regression analysis:

8 $${q}_{N,{b}_{N}}\left(1-\alpha \right)={\gamma }_{0;N,1-\alpha }+{\gamma }_{1;N,1-\alpha }{({b}_{N})}^{-1}+{\nu }_{N;1-\alpha ,{b}_{N}}$$

Finally, we estimate the bias-corrected $(1-\alpha )$-quantile as the OLS predicted value for ${b}_{N}=N$:

9 $${q}_{N}^{BC}\left(1-\alpha \right):={\widehat{\gamma }}_{0;N,1-\alpha }+{\widehat{\gamma }}_{1;N,1-\alpha }{(N)}^{-1}$$

ince ${q}_{N,{b}_{N}}(1-\alpha )$ converges in probability to $q({\eta }_{\infty },1-\alpha )$ and ${({b}_{N})}^{-1}$ converges to zero as $N\to 0$, ${\widehat{\gamma }}_{0;N,1-\alpha }$ converges in probability to $q({\eta }_{\infty },1-\alpha )$ and the asymptotic properties are not affected. However, computational experiments show that the bias-corrected method is more efficient and more powerful in small samples.

## Appendix B: Computational strategy

According to Arvanitis et al. (2019), the test statistic can be written:

10 $${\eta }_{N}=\sqrt{N}\underset{u\in {U}_{2}}{\mathrm{sup}}\left(\underset{\lambda \in\Lambda }{\mathrm{sup}}{}_{{F}_{N}}\left[u\left({X}^{T}\lambda \right)\right]-\underset{\kappa \in \mathrm{\rm K}}{\mathrm{sup}}{}_{{F}_{N}}\left[u\left({X}^{T}\kappa \right)\right]\right),$$

where $\kappa ,\lambda \ge {w}_{min}$ and the minimum weight is set to be greater than 0 (i.e., ${w}_{min}>0$). The term in parentheses is the difference between the solutions to two standard convex optimization problems of maximizing a quasi-concave objective function over a polyhedral feasible set. The analytic complexity of computing ${\eta }_{N}$ stems from the search over all admissible utility functions (${U}_{2}$). However, the utility functions are univariate, normalized, and have a bounded domain ($X$). As a result, we can approximate ${U}_{2}$ with arbitrary accuracy using a finite set of increasing and concave piecewise-linear functions in the following way.

We partition $X$ into ${N}_{1}$ equally spaced values as $\underline{x}={z}_{1}<\dots <{z}_{{N}_{1}}=\overline{x}$, where ${z}_{n}:=\underline{x}+\frac{n-1}{{N}_{1}-1}(\overline{x}-\underline{x})$, $n=1,\dots ,{N}_{1}$; ${N}_{1}\ge 2$. Instead of equal spacing, the partition could also be based on percentiles of the welfare distribution. Similarly, we partition the interval [0,1], as $0<\frac{1}{{N}_{2}-1}<\dots <\frac{{N}_{2}-1}{{N}_{2}-1}<1$, ${N}_{2}\ge 2$. Using this partition, let

11 $$\underset{\_}{{\eta }_{N}}:=\sqrt{N}\underset{u\in \underset{\_}{{U}_{2}}}{\mathrm{sup}}\left(\underset{\lambda \in\Lambda }{\mathrm{sup}}{}_{{F}_{N}}\left[u\left({X}^{T}\lambda \right)\right]-\underset{\kappa \in \mathrm{\rm K}}{\mathrm{sup}}{}_{{F}_{N}}\left[u\left({X}^{T}\kappa \right)\right]\right);$$

12 $$\underset{\_}{{U}_{2}}:=\left\{u\in {C}^{0}:u\left(y\right)=\sum_{n=1}^{{N}_{1}}{w}_{n}r\left(y;{z}_{n}\right):{\varvec{w}}\in W\right\};$$

13 $$W:=\left\{{\varvec{w}}\in {\left\{0,\frac{1}{{N}_{2}-1},\dots ,\frac{{N}_{2}-1}{{N}_{2}-1},1\right\}}^{{N}_{1}}:\sum_{n=1}^{{N}_{1}}{w}_{n}=1\right\}.$$

Every element $u\in \underset{\_}{{U}_{2}}$ consists of at most ${N}_{2}$ linear line segments with knots at ${N}_{1}$ possible outcome levels. Clearly, $\underset{\_}{{U}_{2}}\subset {U}_{2}$ and $\underset{\_}{{\eta }_{N}}$ approximates ${\eta }_{N}$ from below as we refine the partition (${N}_{1},{N}_{2}\to \infty$). The appealing feature of $\underset{\_}{{\eta }_{N}}$ is that we can enumerate all ${N}_{3}:=\frac{1}{{(N}_{1}-1)!}{\prod }_{i=1}^{{N}_{1}-1}({N}_{2}+i-1)$ elements of $\underset{\_}{{U}_{2}}$ for a given partition, and, for every $u\in \underset{\_}{{U}_{2}}$, solve the two embedded maximization problems in (5) using linear programming (LP):

14 $${c}_{0,n}:=\sum_{m=n}^{{N}_{1}}({c}_{1,m+1}-{c}_{1,m}){z}_{m};$$

15 $${c}_{1,n}:=\sum_{m=n}^{{N}_{1}}{w}_{m};$$

16 $$\mathcal{N}:=\left\{n=1,\dots ,{N}_{1}:{w}_{n}>0\right\}\bigcup \left\{{N}_{1}\right\}.$$

For any given $u\in \underset{\_}{{U}_{2}}$, $\underset{\lambda \in\Lambda }{\mathrm{sup}}{}_{{F}_{N}}\left[u\left({X}^{T}\lambda \right)\right]$ is the optimal value of the objective function of the following LP problem in canonical form:

17 $$ \begin{gathered} max\,N^{ - 1} \mathop \sum \limits_{n = 1}^{N} y_{n} \hfill \\ s.t.:y_{n} - c_{1,n} X_{n}^{T} \lambda \le c_{0,n} ,n = 1, \ldots ,N;n \in {\mathcal{N}}; \hfill \\ \mathop \sum \limits_{i = 1}^{M} \lambda_{i} = 1; \hfill \\ \lambda_{i} \ge w_{min} , i = 1, \ldots ,M \hfill \\ y_{n} :free, n = 1, \ldots ,N. \hfill \\ \end{gathered} $$

The LP problem always has a feasible and finite solution and has $\mathcal{O}(N+M)$ variables (where *N* and *M* represent the number of countries and dimensions, respectively) and constraints, making it small for typical data dimensions. Our application in Sect. 4 is based on the number of countries and dimensions (e.g., *M* = 4, *N* = 188 when property rights and civil liberties from the FH are used), and uses small LP problems, which is perfectly manageable with modern-day computer hardware and solver software.

The total run time of all computations for our application amounts to several working days on a standard desktop PC with a 2.93 GHz quad-core Intel i7 processor, 16 GB of RAM and using MATLAB with the external Gurobi Optimizer solver.

## Footnotes

1 Since the choice of weights can be considered as paternalistic (see e.g., Decancq et al., 2015), alternative multidimensional well-being comparisons based on equivalent incomes were offered by Fleurbaey and Blanchet (2013) and Decancq and Schokkaert (2016).

2 See also UN System Task Team on the Post-2015 UN Development Agenda, (2012b) for the detailed importance of governance for the eradication of poverty and inequality, among many other factors.

3 Institutional quality also led to an increased foreign capital flows and foreign direct investment (see e.g., Busse and Hefeker, 2007; Alforo et al., 2008; Papaioannou, 2009; Azemar and Desbordes, 2013; Pinar and Volkan, 2018 among many others), which in turn reinforces economic growth (see, e.g., Borensztein et al., 1998; Haskel et al., 2007).

4 One should note that there has been an on-going discussion on how aid should be allocated that suggests that donors should also take into account other factors beyond good policies (see, e.g., recent discussions on this issue by Bourguignon and Platteau, 2013; Guillaumont et al., 2017). However, our main concern for this paper is that countries with good governance use aid more effectively, and governance promotes a better environment for individuals living in these countries.

5 In this study, we focus on the second-order stochastic dominance (SSD), because the assumptions of non-satiation and risk aversion are generally well accepted in the context of expected utility theory and dual theory of risk of Yaari (1987), and are robust to common transformations of well-being outcomes and probabilities.

6 We use the arithmetic average of the HDI for our analysis since our SD spanning methodology tests whether inclusion of additional institutional quality proxy leads to spanning or not by using a linear optimization problem. However, there is a little difference in HDI scores of countries when composite HDI scores are calculated with the use of arithmetic or geometric mean (e.g., median difference in composite HDI is only 0.004 in 2015) and different ways of calculating final composite index do not affect the qualitative findings of this paper.

7 For instance, we divide the corruption perceptions index by 100.

8 The correlation matrix among the augmented composite indices obtained with respective governance indicators and the the official HDI are positive and highly significant.

9 We find the ranking changes between the augmented index obtained with each governance indicator compared and benchmark HDI in 2015, and then get the average ranking changes of countries between augmented indices that lead to welfare gains (losses) and the HDI index. To obtain average changes, we only used the set of countries that had an overlapping set of information for all governance indicators (i.e., 133 countries are used for ranking comparisons).

10 The rank changes between the augmented index and equally-weighted index are not provided for each proxy scenario to preserve space but are available upon request from authors.
