# Supplementary material for: Stochastic dominance spanning and augmenting the human development index with institutional quality
Source: Ann Oper Res. 2022 Apr 13;315(1):341–69. doi: 10.1007/s10479-022-04656-w (PMC9334449; doi:10.1007/s10479-022-04656-w)
Supplement: Supplementary file 1 — Supplementary file1 (DOCX 175 KB) [file 10479_2022_4656_MOESM1_ESM.docx]

**Supplementary material A. Detailed results of the spanning tests for the inclusion of governance indicators**

| Table S1. Spanning tests for the inclusion of CPI to the HDI | | | | | | | |
| --- | --- | --- | --- | --- | --- | --- | --- |
| Year | Number of countries | Scenario when lower bound weight = 0.10 | | | Scenario when lower bound weight = 0.15 | | |
|  |  | Test Statistic | Critical value (95%) | Result | Test Statistic | Critical value (95%) | Result |
| Panel A. Spanning test results for second-order stochastically dominating scenario with the inclusion of indicator | | | | | | | |
| 2015 | 162 | 0.0000 | 0.0000 | S | 0.0000 | 0.0000 | S |
| 2014 | 170 | 0.0000 | 0.0000 | S | 0.0000 | 0.0000 | S |
| 2013 | 172 | 0.0000 | 0.0000 | S | 0.0000 | 0.0000 | S |
| 2012 | 171 | 0.0000 | 0.0000 | S | 0.0000 | 0.0000 | S |
| 2011 | 178 | 0.0000 | 0.0000 | S | 0.0000 | 0.0000 | S |
| 2010 | 174 | 0.0000 | 0.0000 | S | 0.0000 | 0.0000 | S |
| Panel B. Spanning test results for second-order stochastically dominated scenario with the inclusion of indicator | | | | | | | |
| 2015 | 162 | 1.5853 | 1.2741 | RS | 1.2456 | 1.0024 | RS |
| 2014 | 170 | 1.6576 | 1.2779 | RS | 1.3024 | 1.0041 | RS |
| 2013 | 172 | 1.6752 | 1.2882 | RS | 1.3162 | 1.0122 | RS |
| 2012 | 171 | 1.5998 | 1.2544 | RS | 1.2570 | 0.9856 | RS |
| 2011 | 178 | 1.7865 | 1.4219 | RS | 1.4037 | 1.1173 | RS |
| 2010 | 174 | 1.6717 | 1.3470 | RS | 1.3135 | 1.0584 | RS |
| S: Fail to reject spanning at the 5% level, and RS: Reject spanning at the 5% level | | | | | | | |

| Table S2. Spanning tests for the inclusion of democracy index of the Polity IV to the HDI | | | | | | | |
| --- | --- | --- | --- | --- | --- | --- | --- |
| Year | Number of countries | Scenario when lower bound weight = 0.10 | | | Scenario when lower bound weight = 0.15 | | |
|  |  | Test Statistic | Critical value (95%) | Result | Test Statistic | Critical value (95%) | Result |
| Panel A. Spanning test results for second-order stochastically dominating scenario with the inclusion of indicator | | | | | | | |
| 2015 | 156 | 0.0066 | 0.0046 | RS | 0.0006 | 0.0005 | RS |
| 2014 | 157 | 0.0067 | 0.0044 | RS | 0.0022 | 0.0020 | RS |
| 2013 | 156 | 0.0060 | 0.0040 | RS | 0.0021 | 0.0012 | RS |
| 2012 | 156 | 0.0058 | 0.0046 | RS | 0.0028 | 0.0012 | RS |
| 2011 | 158 | 0.0083 | 0.0073 | RS | 0.0066 | 0.0012 | RS |
| 2010 | 157 | 0.0080 | 0.0012 | RS | 0.0066 | 0.0059 | RS |
| Panel B. Spanning test results for second-order stochastically dominated scenario with the inclusion of indicator | | | | | | | |
| 2015 | 156 | 0.0000 | 0.0000 | S | 0.0000 | 0.0000 | S |
| 2014 | 157 | 0.0000 | 0.0000 | S | 0.0000 | 0.0000 | S |
| 2013 | 156 | 0.0000 | 0.0000 | S | 0.0000 | 0.0000 | S |
| 2012 | 156 | 0.0000 | 0.0000 | S | 0.0000 | 0.0000 | S |
| 2011 | 158 | 0.0000 | 0.0000 | S | 0.0000 | 0.0000 | S |
| 2010 | 157 | 0.0000 | 0.0000 | S | 0.0000 | 0.0000 | S |
| S: Fail to reject spanning at the 5% level, and RS: Reject spanning at the 5% level | | | | | | | |

| Table S3. Spanning tests for the inclusion of property rights of the HF to the HDI | | | | | | | |
| --- | --- | --- | --- | --- | --- | --- | --- |
| Year | Number of countries | Scenario when lower bound weight = 0.10 | | | Scenario when lower bound weight = 0.15 | | |
|  |  | Test Statistic | Critical value (95%) | Result | Test Statistic | Critical value (95%) | Result |
| Panel A. Spanning test results for second-order stochastically dominating scenario with the inclusion of indicator | | | | | | | |
| 2015 | 177 | 0.0000 | 0.0000 | S | 0.0000 | 0.0000 | S |
| 2014 | 177 | 0.0000 | 0.0000 | S | 0.0000 | 0.0000 | S |
| 2013 | 176 | 0.0000 | 0.0000 | S | 0.0000 | 0.0000 | S |
| 2012 | 176 | 0.0000 | 0.0000 | S | 0.0000 | 0.0000 | S |
| 2011 | 176 | 0.0000 | 0.0000 | S | 0.0000 | 0.0000 | S |
| 2010 | 176 | 0.0000 | 0.0000 | S | 0.0000 | 0.0000 | S |
| Panel B. Spanning test results for second-order stochastically dominated scenario with the inclusion of indicator | | | | | | | |
| 2015 | 177 | 1.7586 | 1.3722 | RS | 1.3818 | 1.0024 | RS |
| 2014 | 177 | 1.6129 | 1.2951 | RS | 1.2673 | 1.0176 | RS |
| 2013 | 176 | 1.5502 | 1.2249 | RS | 1.2180 | 0.9626 | RS |
| 2012 | 176 | 1.4815 | 1.1765 | RS | 1.1640 | 0.9250 | RS |
| 2011 | 176 | 1.4030 | 1.1110 | RS | 1.1024 | 0.8738 | RS |
| 2010 | 176 | 1.2950 | 1.0521 | RS | 1.0175 | 0.8266 | RS |
| S: Fail to reject spanning at the 5% level, and RS: Reject spanning at the 5% level | | | | | | | |

| Table S4. Spanning tests for the inclusion of economic freedom component of EFW of the FI | | | | | | | |
| --- | --- | --- | --- | --- | --- | --- | --- |
| Year | Number of countries | Scenario when lower bound weight = 0.10 | | | Scenario when lower bound weight = 0.15 | | |
|  |  | Test Statistic | Critical value (95%) | Result | Test Statistic | Critical value (95%) | Result |
| Panel A. Spanning test results for second-order stochastically dominating scenario with the inclusion of indicator | | | | | | | |
| 2015 | 158 | 0.0243 | 0.0115 | RS | 0.0172 | 0.0129 | RS |
| 2014 | 158 | 0.0285 | 0.0166 | RS | 0.0251 | 0.0133 | RS |
| 2013 | 156 | 0.0360 | 0.0211 | RS | 0.0309 | 0.0098 | RS |
| 2012 | 152 | 0.0361 | 0.0288 | RS | 0.0296 | 0.0125 | RS |
| 2011 | 152 | 0.0453 | 0.0339 | RS | 0.0424 | 0.0326 | RS |
| 2010 | 152 | 0.0570 | 0.0379 | RS | 0.0530 | 0.0440 | RS |
| Panel B. Spanning test results for second-order stochastically dominated scenario with the inclusion of indicator | | | | | | | |
| 2015 | 158 | 0.2090 | 0.2187 | S | 0.1715 | 0.1737 | S |
| 2014 | 158 | 0.2157 | 0.2242 | S | 0.1717 | 0.1738 | S |
| 2013 | 156 | 0.2262 | 0.2329 | S | 0.1835 | 0.1908 | S |
| 2012 | 152 | 0.1898 | 0.1921 | S | 0.1549 | 0.1685 | S |
| 2011 | 152 | 0.1699 | 0.1768 | S | 0.1413 | 0.1449 | S |
| 2010 | 152 | 0.1760 | 0.1795 | S | 0.1414 | 0.1475 | S |
| S: Fail to reject spanning at the 5% level, and RS: Reject spanning at the 5% level | | | | | | | |

| Table S5. Spanning tests for the inclusion of judicial independence component of EFW of the FI | | | | | | | |
| --- | --- | --- | --- | --- | --- | --- | --- |
| Year | Number of countries | Scenario when lower bound weight = 0.10 | | | Scenario when lower bound weight = 0.15 | | |
|  |  | Test Statistic | Critical value (95%) | Result | Test Statistic | Critical value (95%) | Result |
| Panel A. Spanning test results for second-order stochastically dominating scenario with the inclusion of indicator | | | | | | | |
| 2015 | 151 | 0.0000 | 0.0000 | S | 0.0000 | 0.0000 | S |
| 2014 | 150 | 0.0000 | 0.0000 | S | 0.0000 | 0.0000 | S |
| 2013 | 148 | 0.0000 | 0.0000 | S | 0.0000 | 0.0000 | S |
| 2012 | 143 | 0.0000 | 0.0000 | S | 0.0000 | 0.0000 | S |
| 2011 | 142 | 0.0000 | 0.0000 | S | 0.0000 | 0.0000 | S |
| 2010 | 140 | 0.0000 | 0.0000 | S | 0.0000 | 0.0000 | S |
| Panel B. Spanning test results for second-order stochastically dominated scenario with the inclusion of indicator | | | | | | | |
| 2015 | 151 | 1.2568 | 0.9755 | RS | 0.9875 | 0.7668 | RS |
| 2014 | 150 | 1.3366 | 1.0218 | RS | 1.0502 | 0.8029 | RS |
| 2013 | 148 | 1.4026 | 1.1121 | RS | 1.1021 | 0.8735 | RS |
| 2012 | 143 | 1.4422 | 1.0897 | RS | 1.1331 | 0.8565 | RS |
| 2011 | 142 | 1.3848 | 1.0860 | RS | 1.0880 | 0.8537 | RS |
| 2010 | 140 | 1.2658 | 1.0213 | RS | 0.9946 | 0.8024 | RS |
| S: Fail to reject spanning at the 5% level, and RS: Reject spanning at the 5% level | | | | | | | |

| Table S6. Spanning tests for inclusion of the property rights component of EFW of the FI | | | | | | | |
| --- | --- | --- | --- | --- | --- | --- | --- |
| Year | Number of countries | Scenario when lower bound weight = 0.10 | | | Scenario when lower bound weight = 0.15 | | |
|  |  | Test Statistic | Critical value (95%) | Result | Test Statistic | Critical value (95%) | Result |
| Panel A. Spanning test results for second-order stochastically dominating scenario with the inclusion of indicator | | | | | | | |
| 2015 | 151 | 0.0000 | 0.0000 | S | 0.0000 | 0.0000 | S |
| 2014 | 149 | 0.0000 | 0.0000 | S | 0.0000 | 0.0000 | S |
| 2013 | 150 | 0.0000 | 0.0000 | S | 0.0000 | 0.0000 | S |
| 2012 | 143 | 0.0000 | 0.0000 | S | 0.0000 | 0.0000 | S |
| 2011 | 142 | 0.0000 | 0.0000 | S | 0.0000 | 0.0000 | S |
| 2010 | 140 | 0.0000 | 0.0000 | S | 0.0000 | 0.0000 | S |
| Panel B. Spanning test results for second-order stochastically dominated scenario with the inclusion of indicator | | | | | | | |
| 2015 | 151 | 0.7505 | 0.6402 | RS | 0.6119 | 0.5142 | RS |
| 2014 | 149 | 0.8517 | 0.6670 | RS | 0.6709 | 0.5308 | RS |
| 2013 | 150 | 0.9751 | 0.7683 | RS | 0.7661 | 0.6036 | RS |
| 2012 | 143 | 0.8882 | 0.6986 | RS | 0.6979 | 0.5486 | RS |
| 2011 | 142 | 0.7989 | 0.6200 | RS | 0.6332 | 0.4910 | RS |
| 2010 | 140 | 0.7537 | 0.5943 | RS | 0.6017 | 0.4707 | RS |
| S: Fail to reject spanning at the 5% level, and RS: Reject spanning at the 5% level | | | | | | | |

| Table S7. Spanning tests for inclusion of the legal system and property rights component of EFW of the FI | | | | | | | |
| --- | --- | --- | --- | --- | --- | --- | --- |
| Year | Number of countries | Scenario when lower bound weight = 0.10 | | | Scenario when lower bound weight = 0.15 | | |
|  |  | Test Statistic | Critical value (95%) | Result | Test Statistic | Critical value (95%) | Result |
| Panel A. Spanning test results for second-order stochastically dominating scenario with the inclusion of indicator | | | | | | | |
| 2015 | 158 | 0.0000 | 0.0000 | S | 0.0000 | 0.0000 | S |
| 2014 | 158 | 0.0000 | 0.0000 | S | 0.0000 | 0.0000 | S |
| 2013 | 156 | 0.0000 | 0.0000 | S | 0.0000 | 0.0000 | S |
| 2012 | 152 | 0.0000 | 0.0000 | S | 0.0000 | 0.0000 | S |
| 2011 | 152 | 0.0000 | 0.0000 | S | 0.0000 | 0.0000 | S |
| 2010 | 152 | 0.0000 | 0.0000 | S | 0.0000 | 0.0000 | S |
| Panel B. Spanning test results for second-order stochastically dominated scenario with the inclusion of indicator | | | | | | | |
| 2015 | 158 | 0.9646 | 0.7992 | RS | 0.7584 | 0.6295 | RS |
| 2014 | 158 | 0.7779 | 0.6268 | RS | 0.6156 | 0.4955 | RS |
| 2013 | 156 | 0.8051 | 0.6384 | RS | 0.6375 | 0.5078 | RS |
| 2012 | 152 | 0.7542 | 0.6065 | RS | 0.5940 | 0.4778 | RS |
| 2011 | 152 | 0.6692 | 0.5459 | RS | 0.5326 | 0.4323 | RS |
| 2010 | 152 | 0.6098 | 0.4919 | RS | 0.4869 | 0.3925 | RS |
| S: Fail to reject spanning at the 5% level, and RS: Reject spanning at the 5% level | | | | | | | |

| Table S8. Spanning tests for inclusion of the extra payments/bribes/favouritism component of EFW of the FI | | | | | | | |
| --- | --- | --- | --- | --- | --- | --- | --- |
| Year | Number of countries | Scenario when lower bound weight = 0.10 | | | Scenario when lower bound weight = 0.15 | | |
|  |  | Test Statistic | Critical value (95%) | Result | Test Statistic | Critical value (95%) | Result |
| Panel A. Spanning test results for second-order stochastically dominating scenario with the inclusion of indicator | | | | | | | |
| 2015 | 150 | 0.0000 | 0.0000 | S | 0.0000 | 0.0000 | S |
| 2014 | 145 | 0.0000 | 0.0000 | S | 0.0000 | 0.0000 | S |
| 2013 | 148 | 0.0000 | 0.0000 | S | 0.0000 | 0.0000 | S |
| 2012 | 143 | 0.0000 | 0.0000 | S | 0.0000 | 0.0000 | S |
| 2011 | 142 | 0.0000 | 0.0000 | S | 0.0000 | 0.0000 | S |
| 2010 | 140 | 0.0000 | 0.0000 | S | 0.0000 | 0.0000 | S |
| Panel B. Spanning test results for second-order stochastically dominated scenario with the inclusion of indicator | | | | | | | |
| 2015 | 150 | 1.6455 | 1.3461 | RS | 1.2929 | 1.0599 | RS |
| 2014 | 145 | 1.6643 | 1.3160 | RS | 1.3076 | 1.0344 | RS |
| 2013 | 148 | 1.2816 | 0.9853 | RS | 1.0070 | 0.7765 | RS |
| 2012 | 143 | 1.3089 | 1.0019 | RS | 1.0285 | 0.7896 | RS |
| 2011 | 142 | 1.1958 | 0.9434 | RS | 0.9396 | 0.7439 | RS |
| 2010 | 140 | 1.1479 | 0.9166 | RS | 0.9019 | 0.7218 | RS |
| S: Fail to reject spanning at the 5% level, and RS: Reject spanning at the 5% level | | | | | | | |

| Table S9. Spanning tests for inclusion of the regulation component of EFW of the FI | | | | | | | |
| --- | --- | --- | --- | --- | --- | --- | --- |
| Year | Number of countries | Scenario when lower bound weight = 0.10 | | | Scenario when lower bound weight = 0.15 | | |
|  |  | Test Statistic | Critical value (95%) | Result | Test Statistic | Critical value (95%) | Result |
| Panel A. Spanning test results for second-order stochastically dominating scenario with the inclusion of indicator | | | | | | | |
| 2015 | 158 | 0.0391 | 0.0288 | RS | 0.0321 | 0.0233 | RS |
| 2014 | 158 | 0.0778 | 0.0566 | RS | 0.0740 | 0.0344 | RS |
| 2013 | 156 | 0.0824 | 0.0544 | RS | 0.0761 | 0.0243 | RS |
| 2012 | 152 | 0.0971 | 0.0039 | RS | 0.0913 | 0.0855 | RS |
| 2011 | 152 | 0.1185 | 0.0981 | RS | 0.1117 | 0.1029 | RS |
| 2010 | 152 | 0.1385 | 0.0944 | RS | 0.1302 | 0.0855 | RS |
| Panel B. Spanning test results for second-order stochastically dominated scenario with the inclusion of indicator | | | | | | | |
| 2015 | 158 | 0.1232 | 0.1320 | S | 0.1067 | 0.1100 | S |
| 2014 | 158 | 0.2010 | 0.2092 | S | 0.1654 | 0.1657 | S |
| 2013 | 156 | 0.1796 | 0.1836 | S | 0.1481 | 0.1485 | S |
| 2012 | 152 | 0.1588 | 0.1617 | S | 0.1314 | 0.1360 | S |
| 2011 | 152 | 0.1744 | 0.1747 | S | 0.1413 | 0.1428 | S |
| 2010 | 152 | 0.1800 | 0.1857 | S | 0.1479 | 0.1542 | S |
| S: Fail to reject spanning at the 5% level, and RS: Reject spanning at the 5% level | | | | | | | |

| Table S10. Spanning tests for the inclusion of property rights component of the FH to the HDI | | | | | | | |
| --- | --- | --- | --- | --- | --- | --- | --- |
| Year | Number of countries | Scenario when lower bound weight = 0.10 | | | Scenario when lower bound weight = 0.15 | | |
|  |  | Test Statistic | Critical value (95%) | Result | Test Statistic | Critical value (95%) | Result |
| Panel A. Spanning test results for second-order stochastically dominating scenario with the inclusion of indicator | | | | | | | |
| 2015 | 188 | 0.0000 | 0.0074 | S | 0.0000 | 0.0075 | S |
| 2014 | 188 | 0.0000 | 0.0098 | S | 0.0000 | 0.0086 | S |
| 2013 | 188 | 0.0000 | 0.0033 | S | 0.0000 | 0.0085 | S |
| 2012 | 188 | 0.0000 | 0.0129 | S | 0.0000 | 0.0110 | S |
| 2011 | 187 | 0.0000 | 0.0084 | S | 0.0000 | 0.0102 | S |
| 2010 | 187 | 0.0000 | 0.0103 | S | 0.0000 | 0.0096 | S |
| Panel B. Spanning test results for second-order stochastically dominated scenario with the inclusion of indicator | | | | | | | |
| 2015 | 188 | 0.2713 | 0.2080 | RS | 0.2132 | 0.1634 | RS |
| 2014 | 188 | 0.2119 | 0.1342 | RS | 0.1665 | 0.1054 | RS |
| 2013 | 188 | 0.2074 | 0.1603 | RS | 0.1629 | 0.1259 | RS |
| 2012 | 188 | 0.1533 | 0.1067 | RS | 0.1205 | 0.0851 | RS |
| 2011 | 187 | 0.1161 | 0.1271 | S | 0.0912 | 0.1007 | S |
| 2010 | 187 | 0.0331 | 0.1482 | S | 0.0260 | 0.1164 | S |
| S: Fail to reject spanning at the 5% level, and RS: Reject spanning at the 5% level | | | | | | | |

| Table S11. Spanning tests for the inclusion of the civil liberties component of the FH to the HDI | | | | | | | |
| --- | --- | --- | --- | --- | --- | --- | --- |
| Year | Number of countries | Scenario when lower bound weight = 0.10 | | | Scenario when lower bound weight = 0.15 | | |
|  |  | Test Statistic | Critical value (95%) | Result | Test Statistic | Critical value (95%) | Result |
| Panel A. Spanning test results for second-order stochastically dominating scenario with the inclusion of indicator | | | | | | | |
| 2015 | 188 | 0.0000 | 0.0089 | S | 0.0000 | 0.0103 | S |
| 2014 | 188 | 0.0000 | 0.0075 | S | 0.0000 | 0.0103 | S |
| 2013 | 188 | 0.0000 | 0.0046 | S | 0.0000 | 0.0104 | S |
| 2012 | 188 | 0.0000 | 0.0102 | S | 0.0000 | 0.0108 | S |
| 2011 | 187 | 0.0000 | 0.0103 | S | 0.0000 | 0.0056 | S |
| 2010 | 187 | 0.0000 | 0.0094 | S | 0.0000 | 0.0103 | S |
| Panel B. Spanning test results for second-order stochastically dominated scenario with the inclusion of indicator | | | | | | | |
| 2015 | 188 | 0.3187 | 0.2691 | RS | 0.2504 | 0.2124 | RS |
| 2014 | 188 | 0.2371 | 0.2073 | RS | 0.1863 | 0.1628 | RS |
| 2013 | 188 | 0.1654 | 0.1424 | RS | 0.1300 | 0.1178 | RS |
| 2012 | 188 | 0.0979 | 0.1194 | S | 0.0769 | 0.1029 | S |
| 2011 | 187 | 0.0368 | 0.0981 | S | 0.0356 | 0.0987 | S |
| 2010 | 187 | 0.0157 | 0.0812 | S | 0.0158 | 0.0808 | S |
| S: Fail to reject spanning at the 5% level, and RS: Reject spanning at the 5% level | | | | | | | |

| Table S12. Spanning tests for the inclusion of the property rights & civil liberties component of the FH to the HDI | | | | | | | |
| --- | --- | --- | --- | --- | --- | --- | --- |
| Year | Number of countries | Scenario when lower bound weight = 0.10 | | | Scenario when lower bound weight = 0.15 | | |
|  |  | Test Statistic | Critical value (95%) | Result | Test Statistic | Critical value (95%) | Result |
| Panel A. Spanning test results for second-order stochastically dominating scenario with the inclusion of indicator | | | | | | | |
| 2015 | 188 | 0.0000 | 0.0056 | S | 0.0000 | 0.0056 | S |
| 2014 | 188 | 0.0000 | 0.0104 | S | 0.0000 | 0.0039 | S |
| 2013 | 188 | 0.0000 | 0.0087 | S | 0.0000 | 0.0110 | S |
| 2012 | 188 | 0.0000 | 0.0094 | S | 0.0000 | 0.0098 | S |
| 2011 | 187 | 0.0000 | 0.0119 | S | 0.0000 | 0.0049 | S |
| 2010 | 187 | 0.0000 | 0.0103 | S | 0.0000 | 0.0085 | S |
| Panel B. Spanning test results for second-order stochastically dominated scenario with the inclusion of indicator | | | | | | | |
| 2015 | 188 | 0.2998 | 0.2369 | RS | 0.2356 | 0.1862 | RS |
| 2014 | 188 | 0.2270 | 0.1785 | RS | 0.1784 | 0.1406 | RS |
| 2013 | 188 | 0.1824 | 0.1478 | RS | 0.1433 | 0.1170 | RS |
| 2012 | 188 | 0.1202 | 0.1127 | RS | 0.0945 | 0.0907 | RS |
| 2011 | 187 | 0.0475 | 0.1012 | S | 0.0373 | 0.0912 | S |
| 2010 | 187 | 0.0154 | 0.0869 | S | 0.0152 | 0.0779 | S |
| S: Fail to reject spanning at the 5% level, and RS: Reject spanning at the 5% level | | | | | | | |

| Table S13. SD spanning tests for the inclusion of the governance indicator to the HDI when lower bound is set to 0.2 | | | | | | | |
| --- | --- | --- | --- | --- | --- | --- | --- |
| Year | Number of countries | Spanning test results for second-order stochastically dominating scenario with the inclusion of indicator | | | Spanning test results for second-order stochastically dominated scenario with the inclusion of indicator | | |
|  |  | Test Statistic | Critical value (95%) | Result | Test Statistic | Critical value (95%) | Result |
| Panel A. Spanning tests for the inclusion of CPI to the HDI | | | | | | | |
| 2015 | 162 | 0.0000 | 0.0000 | S | 2.6157 | 2.0118 | RS |
| 2014 | 170 | 0.0000 | 0.0000 | S | 2.7351 | 2.0766 | RS |
| 2013 | 172 | 0.0000 | 0.0000 | S | 2.7641 | 2.0845 | RS |
| 2012 | 171 | 0.0000 | 0.0000 | S | 2.6397 | 2.0487 | RS |
| 2011 | 178 | 0.0000 | 0.0000 | S | 2.9478 | 2.2765 | RS |
| 2010 | 174 | 0.0000 | 0.0000 | S | 2.7584 | 2.0859 | RS |
| Panel B. Spanning tests for the inclusion of democracy index of the Polity IV to the HDI | | | | | | | |
| 2015 | 156 | 0.0052 | 0.0049 | RS | 0.0000 | 0.0000 | S |
| 2014 | 157 | 0.0062 | 0.0041 | RS | 0.0000 | 0.0000 | S |
| 2013 | 156 | 0.0058 | 0.0053 | RS | 0.0000 | 0.0000 | S |
| 2012 | 156 | 0.0062 | 0.0049 | RS | 0.0000 | 0.0000 | S |
| 2011 | 158 | 0.0091 | 0.0071 | RS | 0.0000 | 0.0000 | S |
| 2010 | 157 | 0.0081 | 0.0013 | RS | 0.0000 | 0.0000 | S |
| Panel C. Spanning tests for the inclusion of property rights of the HF to the HDI | | | | | | | |
| 2015 | 177 | 0.0000 | 0.0000 | S | 2.9018 | 2.1976 | RS |
| 2014 | 177 | 0.0000 | 0.0000 | S | 2.6612 | 2.0696 | RS |
| 2013 | 176 | 0.0000 | 0.0000 | S | 2.5578 | 1.9734 | RS |
| 2012 | 176 | 0.0000 | 0.0000 | S | 2.4445 | 1.8977 | RS |
| 2011 | 176 | 0.0000 | 0.0000 | S | 2.3149 | 1.8158 | RS |
| 2010 | 176 | 0.0000 | 0.0000 | S | 2.1367 | 1.7151 | RS |
| Panel D. Spanning tests for the inclusion of economic freedom component of EFW of the FI | | | | | | | |
| 2015 | 158 | 0.0223 | 0.0139 | RS | 0.3058 | 0.3204 | S |
| 2014 | 158 | 0.0243 | 0.0172 | RS | 0.3285 | 0.3362 | S |
| 2013 | 156 | 0.0346 | 0.0238 | RS | 0.3493 | 0.3657 | S |
| 2012 | 152 | 0.0334 | 0.0331 | RS | 0.2859 | 0.2993 | S |
| 2011 | 152 | 0.0443 | 0.0396 | RS | 0.2427 | 0.2741 | S |
| 2010 | 152 | 0.0617 | 0.0311 | RS | 0.2581 | 0.2741 | S |
| Panel E. Spanning tests for the inclusion of judicial independence component of EFW of the FI | | | | | | | |
| 2015 | 151 | 0.0000 | 0.0000 | S | 2.0737 | 1.5743 | RS |
| 2014 | 150 | 0.0000 | 0.0000 | S | 2.2053 | 1.6571 | RS |
| 2013 | 148 | 0.0000 | 0.0000 | S | 2.3143 | 1.8095 | RS |
| 2012 | 143 | 0.0000 | 0.0000 | S | 2.3796 | 1.7571 | RS |
| 2011 | 142 | 0.0000 | 0.0000 | S | 2.2849 | 1.7469 | RS |
| 2010 | 140 | 0.0000 | 0.0000 | S | 2.0886 | 1.6264 | RS |
| Panel F. Spanning tests for inclusion of the property rights component of EFW of the FI | | | | | | | |
| 2015 | 151 | 0.0000 | 0.0000 | S | 1.2377 | 0.9879 | RS |
| 2014 | 149 | 0.0000 | 0.0000 | S | 1.4041 | 1.0755 | RS |
| 2013 | 150 | 0.0000 | 0.0000 | S | 1.6088 | 1.2469 | RS |
| 2012 | 143 | 0.0000 | 0.0000 | S | 1.4656 | 1.1354 | RS |
| 2011 | 142 | 0.0000 | 0.0000 | S | 1.3181 | 1.0081 | RS |
| 2010 | 140 | 0.0000 | 0.0000 | S | 1.2436 | 0.9551 | RS |
| Panel G. Spanning tests for inclusion of the legal system and property rights component of EFW of the FI | | | | | | | |
| 2015 | 158 | 0.0000 | 0.0000 | S | 1.5915 | 1.2952 | RS |
| 2014 | 158 | 0.0000 | 0.0000 | S | 1.2644 | 1.0146 | RS |
| 2013 | 156 | 0.0000 | 0.0000 | S | 1.3152 | 1.0390 | RS |
| 2012 | 152 | 0.0000 | 0.0000 | S | 1.2281 | 0.9924 | RS |
| 2011 | 152 | 0.0000 | 0.0000 | S | 1.0783 | 0.8792 | RS |
| 2010 | 152 | 0.0000 | 0.0000 | S | 0.9778 | 0.7815 | RS |
| Panel H. Spanning tests for inclusion of the extra payments/bribes/favouritism component of EFW of the FI | | | | | | | |
| 2015 | 150 | 0.0000 | 0.0000 | S | 2.7151 | 2.1954 | RS |
| 2014 | 145 | 0.0000 | 0.0000 | S | 2.7460 | 2.1183 | RS |
| 2013 | 148 | 0.0000 | 0.0000 | S | 2.1147 | 1.5834 | RS |
| 2012 | 143 | 0.0000 | 0.0000 | S | 2.1597 | 1.6164 | RS |
| 2011 | 142 | 0.0000 | 0.0000 | S | 1.9731 | 1.5213 | RS |
| 2010 | 140 | 0.0000 | 0.0000 | S | 1.8940 | 1.4741 | RS |
| Panel I. Spanning tests for inclusion of the regulation component of EFW of the FI | | | | | | | |
| 2015 | 158 | 0.0433 | 0.0344 | RS | 0.1672 | 0.1765 | S |
| 2014 | 158 | 0.0811 | 0.0627 | RS | 0.2621 | 0.2635 | S |
| 2013 | 156 | 0.0812 | 0.0566 | RS | 0.2341 | 0.2428 | S |
| 2012 | 152 | 0.0955 | 0.0041 | RS | 0.2114 | 0.2240 | S |
| 2011 | 152 | 0.1089 | 0.0955 | RS | 0.2312 | 0.2397 | S |
| 2010 | 152 | 0.1425 | 0.1011 | RS | 0.2373 | 0.2471 | S |
| Panel J. Spanning tests for the inclusion of property rights component of the FH to the HDI | | | | | | | |
| 2015 | 188 | 0.0000 | 0.0079 | S | 0.4477 | 0.3015 | RS |
| 2014 | 188 | 0.0000 | 0.0090 | S | 0.3496 | 0.1954 | RS |
| 2013 | 188 | 0.0000 | 0.0031 | S | 0.3421 | 0.2390 | RS |
| 2012 | 188 | 0.0000 | 0.0129 | S | 0.2530 | 0.1513 | RS |
| 2011 | 187 | 0.0000 | 0.0084 | S | 0.1915 | 0.1788 | RS |
| 2010 | 187 | 0.0000 | 0.0103 | S | 0.0546 | 0.2147 | S |
| Panel K. Spanning tests for the inclusion of the civil liberties component of the FH to the HDI | | | | | | | |
| 2015 | 188 | 0.0000 | 0.0093 | S | 0.5259 | 0.4226 | RS |
| 2014 | 188 | 0.0000 | 0.0081 | S | 0.3912 | 0.3192 | RS |
| 2013 | 188 | 0.0000 | 0.0042 | S | 0.2730 | 0.2074 | RS |
| 2012 | 188 | 0.0000 | 0.0102 | S | 0.1615 | 0.1604 | RS |
| 2011 | 187 | 0.0000 | 0.0103 | S | 0.0441 | 0.1153 | S |
| 2010 | 187 | 0.0000 | 0.0094 | S | 0.1009 | 0.0881 | RS |
| Panel L. Spanning tests for the inclusion of the property rights & civil liberties component of the FH to the HDI | | | | | | | |
| 2015 | 188 | 0.0000 | 0.0070 | S | 0.4947 | 0.3727 | RS |
| 2014 | 188 | 0.0000 | 0.0105 | S | 0.3746 | 0.2703 | RS |
| 2013 | 188 | 0.0000 | 0.0083 | S | 0.3009 | 0.2214 | RS |
| 2012 | 188 | 0.0000 | 0.0094 | S | 0.1984 | 0.1575 | RS |
| 2011 | 187 | 0.0000 | 0.0119 | S | 0.0784 | 0.1339 | S |
| 2010 | 187 | 0.0000 | 0.0103 | S | 0.2408 | 0.1178 | RS |
| S: Fail to reject spanning at the 5% level, and RS: Reject spanning at the 5% level | | | | | | | |

| Table S14. SD spanning tests for the inclusion of the governance indicator to the HDI when lower bound for spanning tests is set to 0.20 and lower bound for SDE weights set to 0.25 | | | | | | | |
| --- | --- | --- | --- | --- | --- | --- | --- |
| Year | Number of countries | Spanning test results for second-order stochastically dominating scenario with the inclusion of indicator | | | Spanning test results for second-order stochastically dominated scenario with the inclusion of indicator | | |
|  |  | Test Statistic | Critical value (95%) | Result | Test Statistic | Critical value (95%) | Result |
| Panel A. Spanning tests for the inclusion of CPI to the HDI | | | | | | | |
| 2015 | 162 | 0.0000 | 0.0000 | S | 2.3251 | 1.7882 | RS |
| 2014 | 170 | 0.0000 | 0.0000 | S | 2.4312 | 1.8458 | RS |
| 2013 | 172 | 0.0000 | 0.0000 | S | 2.4569 | 1.8529 | RS |
| 2012 | 171 | 0.0000 | 0.0000 | S | 2.3464 | 1.8211 | RS |
| 2011 | 178 | 0.0000 | 0.0000 | S | 2.6203 | 2.0236 | RS |
| 2010 | 174 | 0.0000 | 0.0000 | S | 2.4519 | 1.8542 | RS |
| Panel B. Spanning tests for the inclusion of democracy index of the Polity IV to the HDI | | | | | | | |
| 2015 | 156 | 0.0059 | 0.0034 | RS | 0.0000 | 0.0000 | S |
| 2014 | 157 | 0.0071 | 0.0049 | RS | 0.0000 | 0.0000 | S |
| 2013 | 156 | 0.0059 | 0.0051 | RS | 0.0000 | 0.0000 | S |
| 2012 | 156 | 0.0081 | 0.0051 | RS | 0.0000 | 0.0000 | S |
| 2011 | 158 | 0.0091 | 0.0067 | RS | 0.0000 | 0.0000 | S |
| 2010 | 157 | 0.0098 | 0.0023 | RS | 0.0000 | 0.0000 | S |
| Panel C. Spanning tests for the inclusion of property rights of the HF to the HDI | | | | | | | |
| 2015 | 177 | 0.0000 | 0.0000 | S | 2.5793 | 1.9534 | RS |
| 2014 | 177 | 0.0000 | 0.0000 | S | 2.3655 | 1.8397 | RS |
| 2013 | 176 | 0.0000 | 0.0000 | S | 2.2736 | 1.7542 | RS |
| 2012 | 176 | 0.0000 | 0.0000 | S | 2.1729 | 1.6868 | RS |
| 2011 | 176 | 0.0000 | 0.0000 | S | 2.0577 | 1.6140 | RS |
| 2010 | 176 | 0.0000 | 0.0000 | S | 1.8993 | 1.5245 | RS |
| Panel D. Spanning tests for the inclusion of economic freedom component of EFW of the FI | | | | | | | |
| 2015 | 158 | 0.0266 | 0.0197 | RS | 0.2718 | 0.2848 | S |
| 2014 | 158 | 0.0294 | 0.0163 | RS | 0.2920 | 0.2989 | S |
| 2013 | 156 | 0.0401 | 0.0298 | RS | 0.3105 | 0.3250 | S |
| 2012 | 152 | 0.0398 | 0.0314 | RS | 0.2541 | 0.2660 | S |
| 2011 | 152 | 0.0455 | 0.0398 | RS | 0.2157 | 0.2436 | S |
| 2010 | 152 | 0.0712 | 0.0416 | RS | 0.2294 | 0.2436 | S |
| Panel E. Spanning tests for the inclusion of judicial independence component of EFW of the FI | | | | | | | |
| 2015 | 151 | 0.0000 | 0.0000 | S | 1.8433 | 1.3993 | RS |
| 2014 | 150 | 0.0000 | 0.0000 | S | 1.9603 | 1.4730 | RS |
| 2013 | 148 | 0.0000 | 0.0000 | S | 2.0572 | 1.6085 | RS |
| 2012 | 143 | 0.0000 | 0.0000 | S | 2.1152 | 1.5619 | RS |
| 2011 | 142 | 0.0000 | 0.0000 | S | 2.0310 | 1.5528 | RS |
| 2010 | 140 | 0.0000 | 0.0000 | S | 1.8566 | 1.4456 | RS |
| Panel F. Spanning tests for inclusion of the property rights component of EFW of the FI | | | | | | | |
| 2015 | 151 | 0.0000 | 0.0000 | S | 1.1002 | 0.8781 | RS |
| 2014 | 149 | 0.0000 | 0.0000 | S | 1.2481 | 0.9560 | RS |
| 2013 | 150 | 0.0000 | 0.0000 | S | 1.4301 | 1.1084 | RS |
| 2012 | 143 | 0.0000 | 0.0000 | S | 1.3027 | 1.0092 | RS |
| 2011 | 142 | 0.0000 | 0.0000 | S | 1.1717 | 0.8961 | RS |
| 2010 | 140 | 0.0000 | 0.0000 | S | 1.1055 | 0.8490 | RS |
| Panel G. Spanning tests for inclusion of the legal system and property rights component of EFW of the FI | | | | | | | |
| 2015 | 158 | 0.0000 | 0.0000 | S | 1.4146 | 1.1513 | RS |
| 2014 | 158 | 0.0000 | 0.0000 | S | 1.1239 | 0.9018 | RS |
| 2013 | 156 | 0.0000 | 0.0000 | S | 1.1691 | 0.9236 | RS |
| 2012 | 152 | 0.0000 | 0.0000 | S | 1.0916 | 0.8821 | RS |
| 2011 | 152 | 0.0000 | 0.0000 | S | 0.9584 | 0.7815 | RS |
| 2010 | 152 | 0.0000 | 0.0000 | S | 0.8692 | 0.6946 | RS |
| Panel H. Spanning tests for inclusion of the extra payments/bribes/favouritism component of EFW of the FI | | | | | | | |
| 2015 | 150 | 0.0000 | 0.0000 | S | 2.4134 | 1.9515 | RS |
| 2014 | 145 | 0.0000 | 0.0000 | S | 2.4409 | 1.8829 | RS |
| 2013 | 148 | 0.0000 | 0.0000 | S | 1.8797 | 1.4075 | RS |
| 2012 | 143 | 0.0000 | 0.0000 | S | 1.9198 | 1.4368 | RS |
| 2011 | 142 | 0.0000 | 0.0000 | S | 1.7539 | 1.3522 | RS |
| 2010 | 140 | 0.0000 | 0.0000 | S | 1.6836 | 1.3103 | RS |
| Panel I. Spanning tests for inclusion of the regulation component of EFW of the FI | | | | | | | |
| 2015 | 158 | 0.0488 | 0.0422 | RS | 0.1486 | 0.1569 | S |
| 2014 | 158 | 0.0910 | 0.0650 | RS | 0.2330 | 0.2342 | S |
| 2013 | 156 | 0.0867 | 0.0587 | RS | 0.2081 | 0.2158 | S |
| 2012 | 152 | 0.1023 | 0.0067 | RS | 0.1879 | 0.1991 | S |
| 2011 | 152 | 0.1486 | 0.0990 | RS | 0.2055 | 0.2130 | S |
| 2010 | 152 | 0.1655 | 0.1298 | RS | 0.2109 | 0.2196 | S |
| Panel J. Spanning tests for the inclusion of property rights component of the FH to the HDI | | | | | | | |
| 2015 | 188 | 0.0000 | 0.0065 | S | 0.3980 | 0.2680 | RS |
| 2014 | 188 | 0.0000 | 0.0092 | S | 0.3108 | 0.1737 | RS |
| 2013 | 188 | 0.0000 | 0.0041 | S | 0.3041 | 0.2124 | RS |
| 2012 | 188 | 0.0000 | 0.0124 | S | 0.2249 | 0.1345 | RS |
| 2011 | 187 | 0.0000 | 0.0087 | S | 0.1702 | 0.1589 | RS |
| 2010 | 187 | 0.0000 | 0.0120 | S | 0.0485 | 0.1909 | S |
| Panel K. Spanning tests for the inclusion of the civil liberties component of the FH to the HDI | | | | | | | |
| 2015 | 188 | 0.0000 | 0.0074 | S | 0.4675 | 0.3757 | RS |
| 2014 | 188 | 0.0000 | 0.0088 | S | 0.3478 | 0.2838 | RS |
| 2013 | 188 | 0.0000 | 0.0065 | S | 0.2427 | 0.1844 | RS |
| 2012 | 188 | 0.0000 | 0.0124 | S | 0.1436 | 0.1426 | RS |
| 2011 | 187 | 0.0000 | 0.0154 | S | 0.0392 | 0.1025 | S |
| 2010 | 187 | 0.0000 | 0.0067 | S | 0.0897 | 0.0783 | RS |
| Panel L. Spanning tests for the inclusion of the property rights & civil liberties component of the FH to the HDI | | | | | | | |
| 2015 | 188 | 0.0000 | 0.0087 | S | 0.4398 | 0.3313 | RS |
| 2014 | 188 | 0.0000 | 0.0128 | S | 0.3329 | 0.2402 | RS |
| 2013 | 188 | 0.0000 | 0.0092 | S | 0.2675 | 0.1968 | RS |
| 2012 | 188 | 0.0000 | 0.0176 | S | 0.1763 | 0.1400 | RS |
| 2011 | 187 | 0.0000 | 0.0176 | S | 0.0697 | 0.1190 | S |
| 2010 | 187 | 0.0000 | 0.0132 | S | 0.2141 | 0.1047 | RS |
| S: Fail to reject spanning at the 5% level, and RS: Reject spanning at the 5% level | | | | | | | |

| Table S15. SD spanning tests for the inclusion of the governance indicator to the HDI when lower bound for spanning tests is set to 0.15 and lower bound for SDE weights set to 0.25 | | | | | | | |
| --- | --- | --- | --- | --- | --- | --- | --- |
| Year | Number of countries | Spanning test results for second-order stochastically dominating scenario with the inclusion of indicator | | | Spanning test results for second-order stochastically dominated scenario with the inclusion of indicator | | |
|  |  | Test Statistic | Critical value (95%) | Result | Test Statistic | Critical value (95%) | Result |
| Panel A. Spanning tests for the inclusion of CPI to the HDI | | | | | | | |
| 2015 | 162 | 0.0000 | 0.0000 | S | 1.2788 | 1.0291 | RS |
| 2014 | 170 | 0.0000 | 0.0000 | S | 1.3372 | 1.0309 | RS |
| 2013 | 172 | 0.0000 | 0.0000 | S | 1.3513 | 1.0392 | RS |
| 2012 | 171 | 0.0000 | 0.0000 | S | 1.2905 | 1.0119 | RS |
| 2011 | 178 | 0.0000 | 0.0000 | S | 1.4411 | 1.1471 | RS |
| 2010 | 174 | 0.0000 | 0.0000 | S | 1.3485 | 1.0866 | RS |
| Panel B. Spanning tests for the inclusion of democracy index of the Polity IV to the HDI | | | | | | | |
| 2015 | 156 | 0.0094 | 0.0061 | RS | 0.0000 | 0.0000 | S |
| 2014 | 157 | 0.0060 | 0.0056 | RS | 0.0000 | 0.0000 | S |
| 2013 | 156 | 0.0067 | 0.0058 | RS | 0.0000 | 0.0000 | S |
| 2012 | 156 | 0.0074 | 0.0049 | RS | 0.0000 | 0.0000 | S |
| 2011 | 158 | 0.0093 | 0.0069 | RS | 0.0000 | 0.0000 | S |
| 2010 | 157 | 0.0088 | 0.0025 | RS | 0.0000 | 0.0000 | S |
| Panel C. Spanning tests for the inclusion of property rights of the HF to the HDI | | | | | | | |
| 2015 | 177 | 0.0000 | 0.0000 | S | 1.4186 | 1.1074 | RS |
| 2014 | 177 | 0.0000 | 0.0000 | S | 1.3010 | 1.0447 | RS |
| 2013 | 176 | 0.0000 | 0.0000 | S | 1.2505 | 0.9883 | RS |
| 2012 | 176 | 0.0000 | 0.0000 | S | 1.1951 | 0.9497 | RS |
| 2011 | 176 | 0.0000 | 0.0000 | S | 1.1317 | 0.8971 | RS |
| 2010 | 176 | 0.0000 | 0.0000 | S | 1.0446 | 0.8486 | RS |
| Panel D. Spanning tests for the inclusion of economic freedom component of EFW of the FI | | | | | | | |
| 2015 | 158 | 0.0304 | 0.0165 | RS | 0.1760 | 0.1783 | S |
| 2014 | 158 | 0.0333 | 0.0198 | RS | 0.1763 | 0.1784 | S |
| 2013 | 156 | 0.0388 | 0.0254 | RS | 0.1884 | 0.1959 | S |
| 2012 | 152 | 0.0395 | 0.0316 | RS | 0.1591 | 0.1730 | S |
| 2011 | 152 | 0.0496 | 0.0411 | RS | 0.1451 | 0.1488 | S |
| 2010 | 152 | 0.0602 | 0.0422 | RS | 0.1451 | 0.1514 | S |
| Panel E. Spanning tests for the inclusion of judicial independence component of EFW of the FI | | | | | | | |
| 2015 | 151 | 0.0000 | 0.0000 | S | 1.0138 | 0.7872 | RS |
| 2014 | 150 | 0.0000 | 0.0000 | S | 1.0782 | 0.8243 | RS |
| 2013 | 148 | 0.0000 | 0.0000 | S | 1.1314 | 0.8968 | RS |
| 2012 | 143 | 0.0000 | 0.0000 | S | 1.1633 | 0.8793 | RS |
| 2011 | 142 | 0.0000 | 0.0000 | S | 1.1170 | 0.8765 | RS |
| 2010 | 140 | 0.0000 | 0.0000 | S | 1.0211 | 0.8238 | RS |
| Panel F. Spanning tests for inclusion of the property rights component of EFW of the FI | | | | | | | |
| 2015 | 151 | 0.0000 | 0.0000 | S | 0.6282 | 0.5279 | RS |
| 2014 | 149 | 0.0000 | 0.0000 | S | 0.6887 | 0.5450 | RS |
| 2013 | 150 | 0.0000 | 0.0000 | S | 0.7865 | 0.6197 | RS |
| 2012 | 143 | 0.0000 | 0.0000 | S | 0.7165 | 0.5632 | RS |
| 2011 | 142 | 0.0000 | 0.0000 | S | 0.6500 | 0.5041 | RS |
| 2010 | 140 | 0.0000 | 0.0000 | S | 0.6178 | 0.4833 | RS |
| Panel G. Spanning tests for inclusion of the legal system and property rights component of EFW of the FI | | | | | | | |
| 2015 | 158 | 0.0000 | 0.0000 | S | 0.7787 | 0.6463 | RS |
| 2014 | 158 | 0.0000 | 0.0000 | S | 0.6320 | 0.5087 | RS |
| 2013 | 156 | 0.0000 | 0.0000 | S | 0.6544 | 0.5213 | RS |
| 2012 | 152 | 0.0000 | 0.0000 | S | 0.6099 | 0.4905 | RS |
| 2011 | 152 | 0.0000 | 0.0000 | S | 0.5468 | 0.4438 | RS |
| 2010 | 152 | 0.0000 | 0.0000 | S | 0.4998 | 0.4030 | RS |
| Panel H. Spanning tests for inclusion of the extra payments/bribes/favouritism component of EFW of the FI | | | | | | | |
| 2015 | 150 | 0.0000 | 0.0000 | S | 1.3274 | 1.0882 | RS |
| 2014 | 145 | 0.0000 | 0.0000 | S | 1.3425 | 1.0620 | RS |
| 2013 | 148 | 0.0000 | 0.0000 | S | 1.0339 | 0.7972 | RS |
| 2012 | 143 | 0.0000 | 0.0000 | S | 1.0559 | 0.8107 | RS |
| 2011 | 142 | 0.0000 | 0.0000 | S | 0.9646 | 0.7637 | RS |
| 2010 | 140 | 0.0000 | 0.0000 | S | 0.9260 | 0.7410 | RS |
| Panel I. Spanning tests for inclusion of the regulation component of EFW of the FI | | | | | | | |
| 2015 | 158 | 0.0386 | 0.0311 | RS | 0.1095 | 0.1129 | S |
| 2014 | 158 | 0.0851 | 0.0692 | RS | 0.1698 | 0.1701 | S |
| 2013 | 156 | 0.0923 | 0.0741 | RS | 0.1521 | 0.1525 | S |
| 2012 | 152 | 0.1014 | 0.0066 | RS | 0.1349 | 0.1396 | S |
| 2011 | 152 | 0.1454 | 0.0810 | RS | 0.1450 | 0.1466 | S |
| 2010 | 152 | 0.1654 | 0.0910 | RS | 0.1519 | 0.1583 | S |
| Panel J. Spanning tests for the inclusion of property rights component of the FH to the HDI | | | | | | | |
| 2015 | 188 | 0.0000 | 0.0091 | S | 0.2189 | 0.1678 | RS |
| 2014 | 188 | 0.0000 | 0.0099 | S | 0.1709 | 0.1082 | RS |
| 2013 | 188 | 0.0000 | 0.0042 | S | 0.1673 | 0.1293 | RS |
| 2012 | 188 | 0.0000 | 0.0160 | S | 0.1237 | 0.0874 | RS |
| 2011 | 187 | 0.0000 | 0.0079 | S | 0.0936 | 0.1034 | S |
| 2010 | 187 | 0.0000 | 0.0187 | S | 0.0267 | 0.1195 | S |
| Panel K. Spanning tests for the inclusion of the civil liberties component of the FH to the HDI | | | | | | | |
| 2015 | 188 | 0.0000 | 0.0055 | S | 0.2571 | 0.2181 | RS |
| 2014 | 188 | 0.0000 | 0.0073 | S | 0.1913 | 0.1671 | RS |
| 2013 | 188 | 0.0000 | 0.0055 | S | 0.1335 | 0.1209 | RS |
| 2012 | 188 | 0.0000 | 0.0140 | S | 0.0790 | 0.1056 | S |
| 2011 | 187 | 0.0000 | 0.0165 | S | 0.0365 | 0.1013 | S |
| 2010 | 187 | 0.0000 | 0.0056 | S | 0.0163 | 0.0830 | S |
| Panel L. Spanning tests for the inclusion of the property rights & civil liberties component of the FH to the HDI | | | | | | | |
| 2015 | 188 | 0.0000 | 0.0087 | S | 0.2419 | 0.1912 | RS |
| 2014 | 188 | 0.0000 | 0.0165 | S | 0.1831 | 0.1443 | RS |
| 2013 | 188 | 0.0000 | 0.0057 | S | 0.1471 | 0.1201 | RS |
| 2012 | 188 | 0.0000 | 0.0044 | S | 0.0970 | 0.0931 | RS |
| 2011 | 187 | 0.0000 | 0.0144 | S | 0.0383 | 0.0936 | S |
| 2010 | 187 | 0.0000 | 0.0143 | S | 0.0156 | 0.0800 | S |
| S: Fail to reject spanning at the 5% level, and RS: Reject spanning at the 5% level | | | | | | | |

| Table S16. SD spanning tests for the inclusion of the governance indicator to the HDI when lower bound for spanning tests is set to 0.10 and lower bound for SDE weights set to 0.25 | | | | | | | |
| --- | --- | --- | --- | --- | --- | --- | --- |
| Year | Number of countries | Spanning test results for second-order stochastically dominating scenario with the inclusion of indicator | | | Spanning test results for second-order stochastically dominated scenario with the inclusion of indicator | | |
|  |  | Test Statistic | Critical value (95%) | Result | Test Statistic | Critical value (95%) | Result |
| Panel A. Spanning tests for the inclusion of CPI to the HDI | | | | | | | |
| 2015 | 162 | 0.0000 | 0.0000 | S | 1.6275 | 1.3081 | RS |
| 2014 | 170 | 0.0000 | 0.0000 | S | 1.7018 | 1.3120 | RS |
| 2013 | 172 | 0.0000 | 0.0000 | S | 1.7199 | 1.3226 | RS |
| 2012 | 171 | 0.0000 | 0.0000 | S | 1.6425 | 1.2879 | RS |
| 2011 | 178 | 0.0000 | 0.0000 | S | 1.8342 | 1.4598 | RS |
| 2010 | 174 | 0.0000 | 0.0000 | S | 1.7163 | 1.3829 | RS |
| Panel B. Spanning tests for the inclusion of democracy index of the Polity IV to the HDI | | | | | | | |
| 2015 | 156 | 0.0074 | 0.0050 | RS | 0.0000 | 0.0000 | S |
| 2014 | 157 | 0.0079 | 0.0066 | RS | 0.0000 | 0.0000 | S |
| 2013 | 156 | 0.0064 | 0.0050 | RS | 0.0000 | 0.0000 | S |
| 2012 | 156 | 0.0058 | 0.0045 | RS | 0.0000 | 0.0000 | S |
| 2011 | 158 | 0.0090 | 0.0083 | RS | 0.0000 | 0.0000 | S |
| 2010 | 157 | 0.0074 | 0.0021 | RS | 0.0000 | 0.0000 | S |
| Panel C. Spanning tests for the inclusion of property rights of the HF to the HDI | | | | | | | |
| 2015 | 177 | 0.0000 | 0.0000 | S | 1.8055 | 1.4088 | RS |
| 2014 | 177 | 0.0000 | 0.0000 | S | 1.6559 | 1.3296 | RS |
| 2013 | 176 | 0.0000 | 0.0000 | S | 1.5915 | 1.2576 | RS |
| 2012 | 176 | 0.0000 | 0.0000 | S | 1.5210 | 1.2079 | RS |
| 2011 | 176 | 0.0000 | 0.0000 | S | 1.4404 | 1.1406 | RS |
| 2010 | 176 | 0.0000 | 0.0000 | S | 1.3295 | 1.0802 | RS |
| Panel D. Spanning tests for the inclusion of economic freedom component of EFW of the FI | | | | | | | |
| 2015 | 158 | 0.0304 | 0.0194 | RS | 0.2145 | 0.2246 | S |
| 2014 | 158 | 0.0338 | 0.0203 | RS | 0.2214 | 0.2302 | S |
| 2013 | 156 | 0.0391 | 0.0287 | RS | 0.2322 | 0.2391 | S |
| 2012 | 152 | 0.0354 | 0.0307 | RS | 0.1949 | 0.1972 | S |
| 2011 | 152 | 0.0496 | 0.0399 | RS | 0.1744 | 0.1815 | S |
| 2010 | 152 | 0.0569 | 0.0365 | RS | 0.1806 | 0.1843 | S |
| Panel E. Spanning tests for the inclusion of judicial independence component of EFW of the FI | | | | | | | |
| 2015 | 151 | 0.0000 | 0.0000 | S | 1.2903 | 1.0015 | RS |
| 2014 | 150 | 0.0000 | 0.0000 | S | 1.3722 | 1.0490 | RS |
| 2013 | 148 | 0.0000 | 0.0000 | S | 1.4400 | 1.1418 | RS |
| 2012 | 143 | 0.0000 | 0.0000 | S | 1.4806 | 1.1188 | RS |
| 2011 | 142 | 0.0000 | 0.0000 | S | 1.4217 | 1.1150 | RS |
| 2010 | 140 | 0.0000 | 0.0000 | S | 1.2996 | 1.0485 | RS |
| Panel F. Spanning tests for inclusion of the property rights component of EFW of the FI | | | | | | | |
| 2015 | 151 | 0.0000 | 0.0000 | S | 0.7705 | 0.6573 | RS |
| 2014 | 149 | 0.0000 | 0.0000 | S | 0.8744 | 0.6848 | RS |
| 2013 | 150 | 0.0000 | 0.0000 | S | 1.0011 | 0.7888 | RS |
| 2012 | 143 | 0.0000 | 0.0000 | S | 0.9119 | 0.7172 | RS |
| 2011 | 142 | 0.0000 | 0.0000 | S | 0.8202 | 0.6365 | RS |
| 2010 | 140 | 0.0000 | 0.0000 | S | 0.7738 | 0.6101 | RS |
| Panel G. Spanning tests for inclusion of the legal system and property rights component of EFW of the FI | | | | | | | |
| 2015 | 158 | 0.0000 | 0.0000 | S | 0.9903 | 0.8205 | RS |
| 2014 | 158 | 0.0000 | 0.0000 | S | 0.7986 | 0.6435 | RS |
| 2013 | 156 | 0.0000 | 0.0000 | S | 0.8266 | 0.6554 | RS |
| 2012 | 152 | 0.0000 | 0.0000 | S | 0.7743 | 0.6227 | RS |
| 2011 | 152 | 0.0000 | 0.0000 | S | 0.6870 | 0.5605 | RS |
| 2010 | 152 | 0.0000 | 0.0000 | S | 0.6260 | 0.5050 | RS |
| Panel H. Spanning tests for inclusion of the extra payments/bribes/favouritism component of EFW of the FI | | | | | | | |
| 2015 | 150 | 0.0000 | 0.0000 | S | 1.6894 | 1.3820 | RS |
| 2014 | 145 | 0.0000 | 0.0000 | S | 1.7086 | 1.3511 | RS |
| 2013 | 148 | 0.0000 | 0.0000 | S | 1.3158 | 1.0116 | RS |
| 2012 | 143 | 0.0000 | 0.0000 | S | 1.3438 | 1.0286 | RS |
| 2011 | 142 | 0.0000 | 0.0000 | S | 1.2277 | 0.9686 | RS |
| 2010 | 140 | 0.0000 | 0.0000 | S | 1.1785 | 0.9410 | RS |
| Panel I. Spanning tests for inclusion of the regulation component of EFW of the FI | | | | | | | |
| 2015 | 158 | 0.0365 | 0.0298 | RS | 0.1265 | 0.1355 | S |
| 2014 | 158 | 0.0897 | 0.0667 | RS | 0.2063 | 0.2148 | S |
| 2013 | 156 | 0.0899 | 0.0498 | RS | 0.1843 | 0.1885 | S |
| 2012 | 152 | 0.0931 | 0.0045 | RS | 0.1630 | 0.1660 | S |
| 2011 | 152 | 0.1944 | 0.1043 | RS | 0.1791 | 0.1794 | S |
| 2010 | 152 | 0.1476 | 0.0943 | RS | 0.1848 | 0.1907 | S |
| Panel J. Spanning tests for the inclusion of property rights component of the FH to the HDI | | | | | | | |
| 2015 | 188 | 0.0000 | 0.0090 | S | 0.2786 | 0.2135 | RS |
| 2014 | 188 | 0.0000 | 0.0066 | S | 0.2175 | 0.1378 | RS |
| 2013 | 188 | 0.0000 | 0.0051 | S | 0.2129 | 0.1646 | RS |
| 2012 | 188 | 0.0000 | 0.0175 | S | 0.1574 | 0.1095 | RS |
| 2011 | 187 | 0.0000 | 0.0089 | S | 0.1192 | 0.1305 | S |
| 2010 | 187 | 0.0000 | 0.0156 | S | 0.0340 | 0.1522 | S |
| Panel K. Spanning tests for the inclusion of the civil liberties component of the FH to the HDI | | | | | | | |
| 2015 | 188 | 0.0000 | 0.0103 | S | 0.3272 | 0.2763 | RS |
| 2014 | 188 | 0.0000 | 0.0081 | S | 0.2434 | 0.2128 | RS |
| 2013 | 188 | 0.0000 | 0.0065 | S | 0.1699 | 0.1462 | RS |
| 2012 | 188 | 0.0000 | 0.0185 | S | 0.1005 | 0.1226 | S |
| 2011 | 187 | 0.0000 | 0.0123 | S | 0.0378 | 0.1007 | S |
| 2010 | 187 | 0.0000 | 0.0098 | S | 0.0161 | 0.0834 | S |
| Panel L. Spanning tests for the inclusion of the property rights & civil liberties component of the FH to the HDI | | | | | | | |
| 2015 | 188 | 0.0000 | 0.0075 | S | 0.3078 | 0.2432 | RS |
| 2014 | 188 | 0.0000 | 0.0104 | S | 0.2331 | 0.1833 | RS |
| 2013 | 188 | 0.0000 | 0.0099 | S | 0.1872 | 0.1517 | RS |
| 2012 | 188 | 0.0000 | 0.0096 | S | 0.1234 | 0.1157 | RS |
| 2011 | 187 | 0.0000 | 0.0134 | S | 0.0488 | 0.1039 | S |
| 2010 | 187 | 0.0000 | 0.0177 | S | 0.0158 | 0.0892 | S |
| S: Fail to reject spanning at the 5% level, and RS: Reject spanning at the 5% level | | | | | | | |

| Table S17. SD spanning tests for the inclusion of the governance indicator to the HDI when lower bound for spanning tests is set to 0.20 and lower bound for SDE weights set to 0.20 | | | | | | | |
| --- | --- | --- | --- | --- | --- | --- | --- |
| Year | Number of countries | Spanning test results for second-order stochastically dominating scenario with the inclusion of indicator | | | Spanning test results for second-order stochastically dominated scenario with the inclusion of indicator | | |
|  |  | Test Statistic | Critical value (95%) | Result | Test Statistic | Critical value (95%) | Result |
| Panel A. Spanning tests for the inclusion of CPI to the HDI | | | | | | | |
| 2015 | 162 | 0.0000 | 0.0000 | S | 2.4911 | 1.9160 | RS |
| 2014 | 170 | 0.0000 | 0.0000 | S | 2.6048 | 1.9777 | RS |
| 2013 | 172 | 0.0000 | 0.0000 | S | 2.6324 | 1.9853 | RS |
| 2012 | 171 | 0.0000 | 0.0000 | S | 2.5140 | 1.9512 | RS |
| 2011 | 178 | 0.0000 | 0.0000 | S | 2.8074 | 2.1681 | RS |
| 2010 | 174 | 0.0000 | 0.0000 | S | 2.6270 | 1.9866 | RS |
| Panel B. Spanning tests for the inclusion of democracy index of the Polity IV to the HDI | | | | | | | |
| 2015 | 156 | 0.0059 | 0.0034 | RS | 0.0000 | 0.0000 | S |
| 2014 | 157 | 0.0068 | 0.0048 | RS | 0.0000 | 0.0000 | S |
| 2013 | 156 | 0.0051 | 0.0047 | RS | 0.0000 | 0.0000 | S |
| 2012 | 156 | 0.0067 | 0.0044 | RS | 0.0000 | 0.0000 | S |
| 2011 | 158 | 0.0086 | 0.0078 | RS | 0.0000 | 0.0000 | S |
| 2010 | 157 | 0.0083 | 0.0017 | RS | 0.0000 | 0.0000 | S |
| Panel C. Spanning tests for the inclusion of property rights of the HF to the HDI | | | | | | | |
| 2015 | 177 | 0.0000 | 0.0000 | S | 2.7636 | 2.0930 | RS |
| 2014 | 177 | 0.0000 | 0.0000 | S | 2.5345 | 1.9711 | RS |
| 2013 | 176 | 0.0000 | 0.0000 | S | 2.4360 | 1.8795 | RS |
| 2012 | 176 | 0.0000 | 0.0000 | S | 2.3281 | 1.8073 | RS |
| 2011 | 176 | 0.0000 | 0.0000 | S | 2.2047 | 1.7293 | RS |
| 2010 | 176 | 0.0000 | 0.0000 | S | 2.0349 | 1.6334 | RS |
| Panel D. Spanning tests for the inclusion of economic freedom component of EFW of the FI | | | | | | | |
| 2015 | 158 | 0.0266 | 0.0197 | RS | 0.2912 | 0.3051 | S |
| 2014 | 158 | 0.0277 | 0.0198 | RS | 0.3129 | 0.3202 | S |
| 2013 | 156 | 0.0399 | 0.0344 | RS | 0.3327 | 0.3483 | S |
| 2012 | 152 | 0.0376 | 0.0355 | RS | 0.2722 | 0.2850 | S |
| 2011 | 152 | 0.0499 | 0.0411 | RS | 0.2311 | 0.2610 | S |
| 2010 | 152 | 0.0643 | 0.0397 | RS | 0.2458 | 0.2610 | S |
| Panel E. Spanning tests for the inclusion of judicial independence component of EFW of the FI | | | | | | | |
| 2015 | 151 | 0.0000 | 0.0000 | S | 1.9750 | 1.4993 | RS |
| 2014 | 150 | 0.0000 | 0.0000 | S | 2.1003 | 1.5782 | RS |
| 2013 | 148 | 0.0000 | 0.0000 | S | 2.2041 | 1.7234 | RS |
| 2012 | 143 | 0.0000 | 0.0000 | S | 2.2663 | 1.6734 | RS |
| 2011 | 142 | 0.0000 | 0.0000 | S | 2.1761 | 1.6638 | RS |
| 2010 | 140 | 0.0000 | 0.0000 | S | 1.9892 | 1.5489 | RS |
| Panel F. Spanning tests for inclusion of the property rights component of EFW of the FI | | | | | | | |
| 2015 | 151 | 0.0000 | 0.0000 | S | 1.1788 | 0.9408 | RS |
| 2014 | 149 | 0.0000 | 0.0000 | S | 1.3373 | 1.0243 | RS |
| 2013 | 150 | 0.0000 | 0.0000 | S | 1.5322 | 1.1876 | RS |
| 2012 | 143 | 0.0000 | 0.0000 | S | 1.3958 | 1.0813 | RS |
| 2011 | 142 | 0.0000 | 0.0000 | S | 1.2554 | 0.9601 | RS |
| 2010 | 140 | 0.0000 | 0.0000 | S | 1.1844 | 0.9096 | RS |
| Panel G. Spanning tests for inclusion of the legal system and property rights component of EFW of the FI | | | | | | | |
| 2015 | 158 | 0.0000 | 0.0000 | S | 1.5157 | 1.2335 | RS |
| 2014 | 158 | 0.0000 | 0.0000 | S | 1.2042 | 0.9662 | RS |
| 2013 | 156 | 0.0000 | 0.0000 | S | 1.2526 | 0.9896 | RS |
| 2012 | 152 | 0.0000 | 0.0000 | S | 1.1696 | 0.9451 | RS |
| 2011 | 152 | 0.0000 | 0.0000 | S | 1.0269 | 0.8373 | RS |
| 2010 | 152 | 0.0000 | 0.0000 | S | 0.9313 | 0.7443 | RS |
| Panel H. Spanning tests for inclusion of the extra payments/bribes/favouritism component of EFW of the FI | | | | | | | |
| 2015 | 150 | 0.0000 | 0.0000 | S | 2.5858 | 2.0909 | RS |
| 2014 | 145 | 0.0000 | 0.0000 | S | 2.6153 | 2.0174 | RS |
| 2013 | 148 | 0.0000 | 0.0000 | S | 2.0140 | 1.5080 | RS |
| 2012 | 143 | 0.0000 | 0.0000 | S | 2.0569 | 1.5395 | RS |
| 2011 | 142 | 0.0000 | 0.0000 | S | 1.8792 | 1.4488 | RS |
| 2010 | 140 | 0.0000 | 0.0000 | S | 1.8038 | 1.4039 | RS |
| Panel I. Spanning tests for inclusion of the regulation component of EFW of the FI | | | | | | | |
| 2015 | 158 | 0.0488 | 0.0422 | RS | 0.1592 | 0.1681 | S |
| 2014 | 158 | 0.0870 | 0.0660 | RS | 0.2496 | 0.2509 | S |
| 2013 | 156 | 0.0833 | 0.0622 | RS | 0.2230 | 0.2312 | S |
| 2012 | 152 | 0.0988 | 0.0056 | RS | 0.2013 | 0.2133 | S |
| 2011 | 152 | 0.1233 | 0.1044 | RS | 0.2202 | 0.2283 | S |
| 2010 | 152 | 0.1455 | 0.1045 | RS | 0.2260 | 0.2353 | S |
| Panel J. Spanning tests for the inclusion of property rights component of the FH to the HDI | | | | | | | |
| 2015 | 188 | 0.0000 | 0.0065 | S | 0.4264 | 0.2871 | RS |
| 2014 | 188 | 0.0000 | 0.0099 | S | 0.3330 | 0.1861 | RS |
| 2013 | 188 | 0.0000 | 0.0036 | S | 0.3258 | 0.2276 | RS |
| 2012 | 188 | 0.0000 | 0.0125 | S | 0.2410 | 0.1441 | RS |
| 2011 | 187 | 0.0000 | 0.0084 | S | 0.1824 | 0.1703 | RS |
| 2010 | 187 | 0.0000 | 0.0109 | S | 0.0520 | 0.2045 | S |
| Panel K. Spanning tests for the inclusion of the civil liberties component of the FH to the HDI | | | | | | | |
| 2015 | 188 | 0.0000 | 0.0074 | S | 0.5008 | 0.4025 | RS |
| 2014 | 188 | 0.0000 | 0.0078 | S | 0.3726 | 0.3040 | RS |
| 2013 | 188 | 0.0000 | 0.0045 | S | 0.2600 | 0.1976 | RS |
| 2012 | 188 | 0.0000 | 0.0120 | S | 0.1539 | 0.1528 | RS |
| 2011 | 187 | 0.0000 | 0.0128 | S | 0.0420 | 0.1098 | S |
| 2010 | 187 | 0.0000 | 0.0099 | S | 0.0961 | 0.0839 | RS |
| Panel L. Spanning tests for the inclusion of the property rights & civil liberties component of the FH to the HDI | | | | | | | |
| 2015 | 188 | 0.0000 | 0.0087 | S | 0.4712 | 0.3550 | RS |
| 2014 | 188 | 0.0000 | 0.0145 | S | 0.3567 | 0.2574 | RS |
| 2013 | 188 | 0.0000 | 0.0084 | S | 0.2866 | 0.2109 | RS |
| 2012 | 188 | 0.0000 | 0.0123 | S | 0.1889 | 0.1500 | RS |
| 2011 | 187 | 0.0000 | 0.0159 | S | 0.0746 | 0.1275 | S |
| 2010 | 187 | 0.0000 | 0.0106 | S | 0.2294 | 0.1122 | RS |
| S: Fail to reject spanning at the 5% level, and RS: Reject spanning at the 5% level | | | | | | | |

| Table S18. SD spanning tests for the inclusion of the governance indicator to the HDI when lower bound for spanning tests is set to 0.15 and lower bound for SDE weights set to 0.20 | | | | | | | |
| --- | --- | --- | --- | --- | --- | --- | --- |
| Year | Number of countries | Spanning test results for second-order stochastically dominating scenario with the inclusion of indicator | | | Spanning test results for second-order stochastically dominated scenario with the inclusion of indicator | | |
|  |  | Test Statistic | Critical value (95%) | Result | Test Statistic | Critical value (95%) | Result |
| Panel A. Spanning tests for the inclusion of CPI to the HDI | | | | | | | |
| 2015 | 162 | 0.0000 | 0.0000 | S | 1.3701 | 1.1026 | RS |
| 2014 | 170 | 0.0000 | 0.0000 | S | 1.4327 | 1.1045 | RS |
| 2013 | 172 | 0.0000 | 0.0000 | S | 1.4478 | 1.1134 | RS |
| 2012 | 171 | 0.0000 | 0.0000 | S | 1.3827 | 1.0842 | RS |
| 2011 | 178 | 0.0000 | 0.0000 | S | 1.5441 | 1.2290 | RS |
| 2010 | 174 | 0.0000 | 0.0000 | S | 1.4449 | 1.1642 | RS |
| Panel B. Spanning tests for the inclusion of democracy index of the Polity IV to the HDI | | | | | | | |
| 2015 | 156 | 0.0071 | 0.0052 | RS | 0.0000 | 0.0000 | S |
| 2014 | 157 | 0.0057 | 0.0049 | RS | 0.0000 | 0.0000 | S |
| 2013 | 156 | 0.0061 | 0.0054 | RS | 0.0000 | 0.0000 | S |
| 2012 | 156 | 0.0051 | 0.0048 | RS | 0.0000 | 0.0000 | S |
| 2011 | 158 | 0.0088 | 0.0072 | RS | 0.0000 | 0.0000 | S |
| 2010 | 157 | 0.0073 | 0.0017 | RS | 0.0000 | 0.0000 | S |
| Panel C. Spanning tests for the inclusion of property rights of the HF to the HDI | | | | | | | |
| 2015 | 177 | 0.0000 | 0.0000 | S | 1.5200 | 1.1865 | RS |
| 2014 | 177 | 0.0000 | 0.0000 | S | 1.3940 | 1.1194 | RS |
| 2013 | 176 | 0.0000 | 0.0000 | S | 1.3398 | 1.0589 | RS |
| 2012 | 176 | 0.0000 | 0.0000 | S | 1.2804 | 1.0175 | RS |
| 2011 | 176 | 0.0000 | 0.0000 | S | 1.2126 | 0.9612 | RS |
| 2010 | 176 | 0.0000 | 0.0000 | S | 1.1192 | 0.9093 | RS |
| Panel D. Spanning tests for the inclusion of economic freedom component of EFW of the FI | | | | | | | |
| 2015 | 158 | 0.0295 | 0.0145 | RS | 0.1886 | 0.1911 | S |
| 2014 | 158 | 0.0301 | 0.0145 | RS | 0.1889 | 0.1912 | S |
| 2013 | 156 | 0.0334 | 0.0285 | RS | 0.2019 | 0.2099 | S |
| 2012 | 152 | 0.0366 | 0.0306 | RS | 0.1704 | 0.1854 | S |
| 2011 | 152 | 0.0456 | 0.0398 | RS | 0.1554 | 0.1594 | S |
| 2010 | 152 | 0.0594 | 0.0399 | RS | 0.1555 | 0.1623 | S |
| Panel E. Spanning tests for the inclusion of judicial independence component of EFW of the FI | | | | | | | |
| 2015 | 151 | 0.0000 | 0.0000 | S | 1.0863 | 0.8435 | RS |
| 2014 | 150 | 0.0000 | 0.0000 | S | 1.1552 | 0.8832 | RS |
| 2013 | 148 | 0.0000 | 0.0000 | S | 1.2123 | 0.9609 | RS |
| 2012 | 143 | 0.0000 | 0.0000 | S | 1.2464 | 0.9422 | RS |
| 2011 | 142 | 0.0000 | 0.0000 | S | 1.1968 | 0.9391 | RS |
| 2010 | 140 | 0.0000 | 0.0000 | S | 1.0940 | 0.8826 | RS |
| Panel F. Spanning tests for inclusion of the property rights component of EFW of the FI | | | | | | | |
| 2015 | 151 | 0.0000 | 0.0000 | S | 0.6731 | 0.5656 | RS |
| 2014 | 149 | 0.0000 | 0.0000 | S | 0.7379 | 0.5839 | RS |
| 2013 | 150 | 0.0000 | 0.0000 | S | 0.8427 | 0.6640 | RS |
| 2012 | 143 | 0.0000 | 0.0000 | S | 0.7677 | 0.6035 | RS |
| 2011 | 142 | 0.0000 | 0.0000 | S | 0.6965 | 0.5401 | RS |
| 2010 | 140 | 0.0000 | 0.0000 | S | 0.6619 | 0.5178 | RS |
| Panel G. Spanning tests for inclusion of the legal system and property rights component of EFW of the FI | | | | | | | |
| 2015 | 158 | 0.0000 | 0.0000 | S | 0.8343 | 0.6925 | RS |
| 2014 | 158 | 0.0000 | 0.0000 | S | 0.6772 | 0.5451 | RS |
| 2013 | 156 | 0.0000 | 0.0000 | S | 0.7012 | 0.5586 | RS |
| 2012 | 152 | 0.0000 | 0.0000 | S | 0.6534 | 0.5256 | RS |
| 2011 | 152 | 0.0000 | 0.0000 | S | 0.5858 | 0.4755 | RS |
| 2010 | 152 | 0.0000 | 0.0000 | S | 0.5355 | 0.4318 | RS |
| Panel H. Spanning tests for inclusion of the extra payments/bribes/favouritism component of EFW of the FI | | | | | | | |
| 2015 | 150 | 0.0000 | 0.0000 | S | 1.4222 | 1.1659 | RS |
| 2014 | 145 | 0.0000 | 0.0000 | S | 1.4384 | 1.1378 | RS |
| 2013 | 148 | 0.0000 | 0.0000 | S | 1.1077 | 0.8542 | RS |
| 2012 | 143 | 0.0000 | 0.0000 | S | 1.1313 | 0.8686 | RS |
| 2011 | 142 | 0.0000 | 0.0000 | S | 1.0335 | 0.8183 | RS |
| 2010 | 140 | 0.0000 | 0.0000 | S | 0.9921 | 0.7940 | RS |
| Panel I. Spanning tests for inclusion of the regulation component of EFW of the FI | | | | | | | |
| 2015 | 158 | 0.0375 | 0.0275 | RS | 0.1173 | 0.1209 | S |
| 2014 | 158 | 0.0812 | 0.0607 | RS | 0.1819 | 0.1823 | S |
| 2013 | 156 | 0.0867 | 0.0633 | RS | 0.1629 | 0.1634 | S |
| 2012 | 152 | 0.0944 | 0.0042 | RS | 0.1445 | 0.1496 | S |
| 2011 | 152 | 0.1344 | 0.0760 | RS | 0.1554 | 0.1571 | S |
| 2010 | 152 | 0.1433 | 0.0967 | RS | 0.1627 | 0.1696 | S |
| Panel J. Spanning tests for the inclusion of property rights component of the FH to the HDI | | | | | | | |
| 2015 | 188 | 0.0000 | 0.0095 | S | 0.2345 | 0.1797 | RS |
| 2014 | 188 | 0.0000 | 0.0091 | S | 0.1831 | 0.1159 | RS |
| 2013 | 188 | 0.0000 | 0.0035 | S | 0.1792 | 0.1385 | RS |
| 2012 | 188 | 0.0000 | 0.0155 | S | 0.1325 | 0.0936 | RS |
| 2011 | 187 | 0.0000 | 0.0073 | S | 0.1003 | 0.1108 | S |
| 2010 | 187 | 0.0000 | 0.0143 | S | 0.0286 | 0.1280 | S |
| Panel K. Spanning tests for the inclusion of the civil liberties component of the FH to the HDI | | | | | | | |
| 2015 | 188 | 0.0000 | 0.0081 | S | 0.2755 | 0.2336 | RS |
| 2014 | 188 | 0.0000 | 0.0081 | S | 0.2049 | 0.1791 | RS |
| 2013 | 188 | 0.0000 | 0.0049 | S | 0.1430 | 0.1296 | RS |
| 2012 | 188 | 0.0000 | 0.0121 | S | 0.0846 | 0.1132 | S |
| 2011 | 187 | 0.0000 | 0.0155 | S | 0.0391 | 0.1086 | S |
| 2010 | 187 | 0.0000 | 0.0096 | S | 0.0174 | 0.0889 | S |
| Panel L. Spanning tests for the inclusion of the property rights & civil liberties component of the FH to the HDI | | | | | | | |
| 2015 | 188 | 0.0000 | 0.0077 | S | 0.2591 | 0.2048 | RS |
| 2014 | 188 | 0.0000 | 0.0143 | S | 0.1962 | 0.1547 | RS |
| 2013 | 188 | 0.0000 | 0.0063 | S | 0.1576 | 0.1287 | RS |
| 2012 | 188 | 0.0000 | 0.0056 | S | 0.1039 | 0.0998 | RS |
| 2011 | 187 | 0.0000 | 0.0123 | S | 0.0411 | 0.1003 | S |
| 2010 | 187 | 0.0000 | 0.0115 | S | 0.0167 | 0.0857 | S |
| S: Fail to reject spanning at the 5% level, and RS: Reject spanning at the 5% level | | | | | | | |

| Table S19. SD spanning tests for the inclusion of the governance indicator to the HDI when lower bound for spanning tests is set to 0.10 and lower bound for SDE weights set to 0.20 | | | | | | | |
| --- | --- | --- | --- | --- | --- | --- | --- |
| Year | Number of countries | Spanning test results for second-order stochastically dominating scenario with the inclusion of indicator | | | Spanning test results for second-order stochastically dominated scenario with the inclusion of indicator | | |
|  |  | Test Statistic | Critical value (95%) | Result | Test Statistic | Critical value (95%) | Result |
| Panel A. Spanning tests for the inclusion of CPI to the HDI | | | | | | | |
| 2015 | 162 | 0.0000 | 0.0000 | S | 1.7438 | 1.4015 | RS |
| 2014 | 170 | 0.0000 | 0.0000 | S | 1.8234 | 1.4057 | RS |
| 2013 | 172 | 0.0000 | 0.0000 | S | 1.8427 | 1.4170 | RS |
| 2012 | 171 | 0.0000 | 0.0000 | S | 1.7598 | 1.3798 | RS |
| 2011 | 178 | 0.0000 | 0.0000 | S | 1.9652 | 1.5641 | RS |
| 2010 | 174 | 0.0000 | 0.0000 | S | 1.8389 | 1.4817 | RS |
| Panel B. Spanning tests for the inclusion of democracy index of the Polity IV to the HDI | | | | | | | |
| 2015 | 156 | 0.0064 | 0.0049 | RS | 0.0000 | 0.0000 | S |
| 2014 | 157 | 0.0071 | 0.0051 | RS | 0.0000 | 0.0000 | S |
| 2013 | 156 | 0.0056 | 0.0035 | RS | 0.0000 | 0.0000 | S |
| 2012 | 156 | 0.0051 | 0.0044 | RS | 0.0000 | 0.0000 | S |
| 2011 | 158 | 0.0081 | 0.0079 | RS | 0.0000 | 0.0000 | S |
| 2010 | 157 | 0.0071 | 0.0013 | RS | 0.0000 | 0.0000 | S |
| Panel C. Spanning tests for the inclusion of property rights of the HF to the HDI | | | | | | | |
| 2015 | 177 | 0.0000 | 0.0000 | S | 1.9345 | 1.5094 | RS |
| 2014 | 177 | 0.0000 | 0.0000 | S | 1.7742 | 1.4246 | RS |
| 2013 | 176 | 0.0000 | 0.0000 | S | 1.7052 | 1.3474 | RS |
| 2012 | 176 | 0.0000 | 0.0000 | S | 1.6297 | 1.2942 | RS |
| 2011 | 176 | 0.0000 | 0.0000 | S | 1.5433 | 1.2221 | RS |
| 2010 | 176 | 0.0000 | 0.0000 | S | 1.4245 | 1.1573 | RS |
| Panel D. Spanning tests for the inclusion of economic freedom component of EFW of the FI | | | | | | | |
| 2015 | 158 | 0.0203 | 0.0117 | RS | 0.2298 | 0.2406 | S |
| 2014 | 158 | 0.0279 | 0.0177 | RS | 0.2372 | 0.2466 | S |
| 2013 | 156 | 0.0377 | 0.0234 | RS | 0.2488 | 0.2562 | S |
| 2012 | 152 | 0.0302 | 0.0291 | RS | 0.2088 | 0.2113 | S |
| 2011 | 152 | 0.0468 | 0.0411 | RS | 0.1869 | 0.1945 | S |
| 2010 | 152 | 0.0561 | 0.0349 | RS | 0.1935 | 0.1975 | S |
| Panel E. Spanning tests for the inclusion of judicial independence component of EFW of the FI | | | | | | | |
| 2015 | 151 | 0.0000 | 0.0000 | S | 1.3825 | 1.0731 | RS |
| 2014 | 150 | 0.0000 | 0.0000 | S | 1.4702 | 1.1240 | RS |
| 2013 | 148 | 0.0000 | 0.0000 | S | 1.5429 | 1.2233 | RS |
| 2012 | 143 | 0.0000 | 0.0000 | S | 1.5864 | 1.1987 | RS |
| 2011 | 142 | 0.0000 | 0.0000 | S | 1.5232 | 1.1946 | RS |
| 2010 | 140 | 0.0000 | 0.0000 | S | 1.3924 | 1.1234 | RS |
| Panel F. Spanning tests for inclusion of the property rights component of EFW of the FI | | | | | | | |
| 2015 | 151 | 0.0000 | 0.0000 | S | 0.8255 | 0.7042 | RS |
| 2014 | 149 | 0.0000 | 0.0000 | S | 0.9368 | 0.7337 | RS |
| 2013 | 150 | 0.0000 | 0.0000 | S | 1.0726 | 0.8451 | RS |
| 2012 | 143 | 0.0000 | 0.0000 | S | 0.9771 | 0.7685 | RS |
| 2011 | 142 | 0.0000 | 0.0000 | S | 0.8788 | 0.6820 | RS |
| 2010 | 140 | 0.0000 | 0.0000 | S | 0.8291 | 0.6537 | RS |
| Panel G. Spanning tests for inclusion of the legal system and property rights component of EFW of the FI | | | | | | | |
| 2015 | 158 | 0.0000 | 0.0000 | S | 1.0610 | 0.8791 | RS |
| 2014 | 158 | 0.0000 | 0.0000 | S | 0.8557 | 0.6895 | RS |
| 2013 | 156 | 0.0000 | 0.0000 | S | 0.8856 | 0.7022 | RS |
| 2012 | 152 | 0.0000 | 0.0000 | S | 0.8297 | 0.6672 | RS |
| 2011 | 152 | 0.0000 | 0.0000 | S | 0.7361 | 0.6005 | RS |
| 2010 | 152 | 0.0000 | 0.0000 | S | 0.6708 | 0.5411 | RS |
| Panel H. Spanning tests for inclusion of the extra payments/bribes/favouritism component of EFW of the FI | | | | | | | |
| 2015 | 150 | 0.0000 | 0.0000 | S | 1.8101 | 1.4807 | RS |
| 2014 | 145 | 0.0000 | 0.0000 | S | 1.8307 | 1.4476 | RS |
| 2013 | 148 | 0.0000 | 0.0000 | S | 1.4098 | 1.0838 | RS |
| 2012 | 143 | 0.0000 | 0.0000 | S | 1.4398 | 1.1021 | RS |
| 2011 | 142 | 0.0000 | 0.0000 | S | 1.3154 | 1.0377 | RS |
| 2010 | 140 | 0.0000 | 0.0000 | S | 1.2627 | 1.0083 | RS |
| Panel I. Spanning tests for inclusion of the regulation component of EFW of the FI | | | | | | | |
| 2015 | 158 | 0.0304 | 0.0239 | RS | 0.1355 | 0.1452 | S |
| 2014 | 158 | 0.0803 | 0.0622 | RS | 0.2211 | 0.2301 | S |
| 2013 | 156 | 0.0856 | 0.0587 | RS | 0.1975 | 0.2020 | S |
| 2012 | 152 | 0.0957 | 0.0034 | RS | 0.1747 | 0.1779 | S |
| 2011 | 152 | 0.1899 | 0.0988 | RS | 0.1919 | 0.1922 | S |
| 2010 | 152 | 0.1422 | 0.0933 | RS | 0.1980 | 0.2043 | S |
| Panel J. Spanning tests for the inclusion of property rights component of the FH to the HDI | | | | | | | |
| 2015 | 188 | 0.0000 | 0.0083 | S | 0.2985 | 0.2288 | RS |
| 2014 | 188 | 0.0000 | 0.0091 | S | 0.2331 | 0.1476 | RS |
| 2013 | 188 | 0.0000 | 0.0055 | S | 0.2281 | 0.1763 | RS |
| 2012 | 188 | 0.0000 | 0.0189 | S | 0.1687 | 0.1174 | RS |
| 2011 | 187 | 0.0000 | 0.0083 | S | 0.1277 | 0.1398 | S |
| 2010 | 187 | 0.0000 | 0.0103 | S | 0.0364 | 0.1630 | S |
| Panel K. Spanning tests for the inclusion of the civil liberties component of the FH to the HDI | | | | | | | |
| 2015 | 188 | 0.0000 | 0.0091 | S | 0.3506 | 0.2960 | RS |
| 2014 | 188 | 0.0000 | 0.0087 | S | 0.2608 | 0.2280 | RS |
| 2013 | 188 | 0.0000 | 0.0049 | S | 0.1820 | 0.1566 | RS |
| 2012 | 188 | 0.0000 | 0.0142 | S | 0.1077 | 0.1313 | S |
| 2011 | 187 | 0.0000 | 0.0112 | S | 0.0405 | 0.1079 | S |
| 2010 | 187 | 0.0000 | 0.0094 | S | 0.0173 | 0.0893 | S |
| Panel L. Spanning tests for the inclusion of the property rights & civil liberties component of the FH to the HDI | | | | | | | |
| 2015 | 188 | 0.0000 | 0.0053 | S | 0.3298 | 0.2606 | RS |
| 2014 | 188 | 0.0000 | 0.0103 | S | 0.2497 | 0.1964 | RS |
| 2013 | 188 | 0.0000 | 0.0091 | S | 0.2006 | 0.1626 | RS |
| 2012 | 188 | 0.0000 | 0.0111 | S | 0.1322 | 0.1240 | RS |
| 2011 | 187 | 0.0000 | 0.0128 | S | 0.0523 | 0.1113 | S |
| 2010 | 187 | 0.0000 | 0.0103 | S | 0.0169 | 0.0956 | S |
| S: Fail to reject spanning at the 5% level, and RS: Reject spanning at the 5% level | | | | | | | |

**Supplementary material B. Combinations of components where the spanning is rejected**

| Table S20. Combinations of components where the spanning is rejected for the dominating scenario when lower bound weight is set to 0.10 | | | | | |
| --- | --- | --- | --- | --- | --- |
| Year | Included governance proxy | Health | Education | Income | Governance |
| 2015 | Polity IV - Democracy | 0.695 | 0.100 | 0.100 | 0.105 |
| 2014 | Polity IV - Democracy | 0.700 | 0.100 | 0.100 | 0.100 |
| 2013 | Polity IV - Democracy | 0.700 | 0.100 | 0.100 | 0.100 |
| 2012 | Polity IV - Democracy | 0.670 | 0.100 | 0.100 | 0.130 |
| 2011 | Polity IV - Democracy | 0.667 | 0.100 | 0.100 | 0.133 |
| 2010 | Polity IV - Democracy | 0.681 | 0.100 | 0.100 | 0.119 |
| 2015 | FI – Economic Freedom | 0.344 | 0.100 | 0.100 | 0.456 |
| 2014 | FI – Economic Freedom | 0.332 | 0.100 | 0.100 | 0.468 |
| 2013 | FI – Economic Freedom | 0.280 | 0.100 | 0.100 | 0.520 |
| 2012 | FI – Economic Freedom | 0.253 | 0.100 | 0.100 | 0.547 |
| 2011 | FI – Economic Freedom | 0.273 | 0.100 | 0.100 | 0.527 |
| 2010 | FI – Economic Freedom | 0.249 | 0.100 | 0.100 | 0.551 |
| 2015 | FI – Regulation | 0.397 | 0.100 | 0.100 | 0.403 |
| 2014 | FI – Regulation | 0.289 | 0.100 | 0.100 | 0.511 |
| 2013 | FI – Regulation | 0.315 | 0.100 | 0.100 | 0.485 |
| 2012 | FI – Regulation | 0.259 | 0.100 | 0.100 | 0.541 |
| 2011 | FI – Regulation | 0.229 | 0.100 | 0.100 | 0.571 |
| 2010 | FI – Regulation | 0.186 | 0.100 | 0.100 | 0.614 |

| Table S21. Combinations of components where the spanning is rejected for the dominating scenario when lower bound weight is set to 0.15 | | | | | |
| --- | --- | --- | --- | --- | --- |
| Year | Included governance proxy | Health | Education | Income | Governance |
| 2015 | Polity IV - Democracy | 0.550 | 0.150 | 0.150 | 0.150 |
| 2014 | Polity IV - Democracy | 0.550 | 0.150 | 0.150 | 0.150 |
| 2013 | Polity IV - Democracy | 0.550 | 0.150 | 0.150 | 0.150 |
| 2012 | Polity IV - Democracy | 0.550 | 0.150 | 0.150 | 0.150 |
| 2011 | Polity IV - Democracy | 0.550 | 0.150 | 0.150 | 0.150 |
| 2010 | Polity IV - Democracy | 0.550 | 0.150 | 0.150 | 0.150 |
| 2015 | FI – Economic Freedom | 0.299 | 0.150 | 0.150 | 0.401 |
| 2014 | FI – Economic Freedom | 0.296 | 0.150 | 0.150 | 0.404 |
| 2013 | FI – Economic Freedom | 0.198 | 0.150 | 0.150 | 0.502 |
| 2012 | FI – Economic Freedom | 0.265 | 0.150 | 0.150 | 0.435 |
| 2011 | FI – Economic Freedom | 0.183 | 0.150 | 0.150 | 0.517 |
| 2010 | FI – Economic Freedom | 0.150 | 0.150 | 0.150 | 0.550 |
| 2015 | FI – Regulation | 0.322 | 0.150 | 0.150 | 0.378 |
| 2014 | FI – Regulation | 0.234 | 0.150 | 0.150 | 0.466 |
| 2013 | FI – Regulation | 0.248 | 0.150 | 0.150 | 0.452 |
| 2012 | FI – Regulation | 0.180 | 0.150 | 0.150 | 0.520 |
| 2011 | FI – Regulation | 0.182 | 0.150 | 0.150 | 0.518 |
| 2010 | FI – Regulation | 0.150 | 0.150 | 0.150 | 0.550 |

| Table S22. Combinations of components where the spanning is rejected for the dominated scenario when lower bound weight is set to 0.10 | | | | | |
| --- | --- | --- | --- | --- | --- |
| Year | Included governance proxy | Health | Education | Income | Governance |
| 2015 | CPI | 0.1 | 0.1 | 0.1 | 0.7 |
| 2014 | CPI | 0.1 | 0.1 | 0.1 | 0.7 |
| 2013 | CPI | 0.1 | 0.1 | 0.1 | 0.7 |
| 2012 | CPI | 0.1 | 0.1 | 0.1 | 0.7 |
| 2011 | CPI | 0.1 | 0.1 | 0.1 | 0.7 |
| 2010 | CPI | 0.1 | 0.1 | 0.1 | 0.7 |
| 2015 | HF - PR | 0.1 | 0.1 | 0.1 | 0.7 |
| 2014 | HF - PR | 0.1 | 0.1 | 0.1 | 0.7 |
| 2013 | HF - PR | 0.1 | 0.1 | 0.1 | 0.7 |
| 2012 | HF - PR | 0.1 | 0.1 | 0.1 | 0.7 |
| 2011 | HF - PR | 0.1 | 0.1 | 0.1 | 0.7 |
| 2010 | HF - PR | 0.1 | 0.1 | 0.1 | 0.7 |
| 2015 | FI - Judicial | 0.1 | 0.1 | 0.1 | 0.7 |
| 2014 | FI - Judicial | 0.1 | 0.1 | 0.1 | 0.7 |
| 2013 | FI - Judicial | 0.1 | 0.1 | 0.1 | 0.7 |
| 2012 | FI - Judicial | 0.1 | 0.1 | 0.1 | 0.7 |
| 2011 | FI - Judicial | 0.1 | 0.1 | 0.1 | 0.7 |
| 2010 | FI - Judicial | 0.1 | 0.1 | 0.1 | 0.7 |
| 2015 | FI - PR | 0.1 | 0.1 | 0.1 | 0.7 |
| 2014 | FI - PR | 0.1 | 0.1 | 0.1 | 0.7 |
| 2013 | FI - PR | 0.1 | 0.1 | 0.1 | 0.7 |
| 2012 | FI - PR | 0.1 | 0.1 | 0.1 | 0.7 |
| 2011 | FI - PR | 0.1 | 0.1 | 0.1 | 0.7 |
| 2010 | FI - PR | 0.1 | 0.1 | 0.1 | 0.7 |
| 2015 | FI - LSPR | 0.1 | 0.1 | 0.1 | 0.7 |
| 2014 | FI - LSPR | 0.1 | 0.1 | 0.1 | 0.7 |
| 2013 | FI - LSPR | 0.1 | 0.1 | 0.1 | 0.7 |
| 2012 | FI - LSPR | 0.1 | 0.1 | 0.1 | 0.7 |
| 2011 | FI - LSPR | 0.1 | 0.1 | 0.1 | 0.7 |
| 2010 | FI - LSPR | 0.1 | 0.1 | 0.1 | 0.7 |
| 2015 | FI - Bribes | 0.1 | 0.1 | 0.1 | 0.7 |
| 2014 | FI - Bribes | 0.1 | 0.1 | 0.1 | 0.7 |
| 2013 | FI - Bribes | 0.1 | 0.1 | 0.1 | 0.7 |
| 2012 | FI - Bribes | 0.1 | 0.1 | 0.1 | 0.7 |
| 2011 | FI - Bribes | 0.1 | 0.1 | 0.1 | 0.7 |
| 2010 | FI - Bribes | 0.1 | 0.1 | 0.1 | 0.7 |
| 2015 | FH - PR | 0.1 | 0.1 | 0.1 | 0.7 |
| 2014 | FH - PR | 0.1 | 0.1 | 0.1 | 0.7 |
| 2013 | FH - PR | 0.1 | 0.1 | 0.1 | 0.7 |
| 2012 | FH - PR | 0.1 | 0.1 | 0.1 | 0.7 |
| 2015 | FH - CL | 0.1 | 0.1 | 0.1 | 0.7 |
| 2014 | FH - CL | 0.1 | 0.1 | 0.1 | 0.7 |
| 2013 | FH - CL | 0.1 | 0.1 | 0.1 | 0.7 |
| 2015 | FH - PRCL | 0.1 | 0.1 | 0.1 | 0.7 |
| 2014 | FH - PRCL | 0.1 | 0.1 | 0.1 | 0.7 |
| 2013 | FH - PRCL | 0.1 | 0.1 | 0.1 | 0.7 |
| 2012 | FH - PRCL | 0.1 | 0.1 | 0.1 | 0.7 |

| Table S23. Combinations of components where the spanning is rejected for the dominated scenario when lower bound weight is set to 0.15 | | | | | |
| --- | --- | --- | --- | --- | --- |
| Year | Included governance proxy | Health | Education | Income | Governance |
| 2015 | CPI | 0.15 | 0.15 | 0.15 | 0.55 |
| 2014 | CPI | 0.15 | 0.15 | 0.15 | 0.55 |
| 2013 | CPI | 0.15 | 0.15 | 0.15 | 0.55 |
| 2012 | CPI | 0.15 | 0.15 | 0.15 | 0.55 |
| 2011 | CPI | 0.15 | 0.15 | 0.15 | 0.55 |
| 2010 | CPI | 0.15 | 0.15 | 0.15 | 0.55 |
| 2015 | HF - PR | 0.15 | 0.15 | 0.15 | 0.55 |
| 2014 | HF - PR | 0.15 | 0.15 | 0.15 | 0.55 |
| 2013 | HF - PR | 0.15 | 0.15 | 0.15 | 0.55 |
| 2012 | HF - PR | 0.15 | 0.15 | 0.15 | 0.55 |
| 2011 | HF - PR | 0.15 | 0.15 | 0.15 | 0.55 |
| 2010 | HF - PR | 0.15 | 0.15 | 0.15 | 0.55 |
| 2015 | FI - Judicial | 0.15 | 0.15 | 0.15 | 0.55 |
| 2014 | FI - Judicial | 0.15 | 0.15 | 0.15 | 0.55 |
| 2013 | FI - Judicial | 0.15 | 0.15 | 0.15 | 0.55 |
| 2012 | FI - Judicial | 0.15 | 0.15 | 0.15 | 0.55 |
| 2011 | FI - Judicial | 0.15 | 0.15 | 0.15 | 0.55 |
| 2010 | FI - Judicial | 0.15 | 0.15 | 0.15 | 0.55 |
| 2015 | FI - PR | 0.15 | 0.15 | 0.15 | 0.55 |
| 2014 | FI - PR | 0.15 | 0.15 | 0.15 | 0.55 |
| 2013 | FI - PR | 0.15 | 0.15 | 0.15 | 0.55 |
| 2012 | FI - PR | 0.15 | 0.15 | 0.15 | 0.55 |
| 2011 | FI - PR | 0.15 | 0.15 | 0.15 | 0.55 |
| 2010 | FI - PR | 0.15 | 0.15 | 0.15 | 0.55 |
| 2015 | FI - LSPR | 0.15 | 0.15 | 0.15 | 0.55 |
| 2014 | FI - LSPR | 0.15 | 0.15 | 0.15 | 0.55 |
| 2013 | FI - LSPR | 0.15 | 0.15 | 0.15 | 0.55 |
| 2012 | FI - LSPR | 0.15 | 0.15 | 0.15 | 0.55 |
| 2011 | FI - LSPR | 0.15 | 0.15 | 0.15 | 0.55 |
| 2010 | FI - LSPR | 0.15 | 0.15 | 0.15 | 0.55 |
| 2015 | FI - Bribes | 0.15 | 0.15 | 0.15 | 0.55 |
| 2014 | FI - Bribes | 0.15 | 0.15 | 0.15 | 0.55 |
| 2013 | FI - Bribes | 0.15 | 0.15 | 0.15 | 0.55 |
| 2012 | FI - Bribes | 0.15 | 0.15 | 0.15 | 0.55 |
| 2011 | FI - Bribes | 0.15 | 0.15 | 0.15 | 0.55 |
| 2010 | FI - Bribes | 0.15 | 0.15 | 0.15 | 0.55 |
| 2015 | FH - PR | 0.15 | 0.15 | 0.15 | 0.55 |
| 2014 | FH - PR | 0.15 | 0.15 | 0.15 | 0.55 |
| 2013 | FH - PR | 0.15 | 0.15 | 0.15 | 0.55 |
| 2012 | FH - PR | 0.15 | 0.15 | 0.15 | 0.55 |
| 2015 | FH - CL | 0.15 | 0.15 | 0.15 | 0.55 |
| 2014 | FH - CL | 0.15 | 0.15 | 0.15 | 0.55 |
| 2013 | FH - CL | 0.15 | 0.15 | 0.15 | 0.55 |
| 2015 | FH - PRCL | 0.15 | 0.15 | 0.15 | 0.55 |
| 2014 | FH - PRCL | 0.15 | 0.15 | 0.15 | 0.55 |
| 2013 | FH - PRCL | 0.15 | 0.15 | 0.15 | 0.55 |
| 2012 | FH - PRCL | 0.15 | 0.15 | 0.15 | 0.55 |

| Table S24. Combinations of components where the spanning is rejected for the dominating scenario when lower bound weight is set to 0.20 | | | | | |
| --- | --- | --- | --- | --- | --- |
| Year | Included governance proxy | Health | Education | Income | Governance |
| 2015 | Polity IV - Democracy | 0.400 | 0.200 | 0.200 | 0.200 |
| 2014 | Polity IV - Democracy | 0.400 | 0.200 | 0.200 | 0.200 |
| 2013 | Polity IV - Democracy | 0.400 | 0.200 | 0.200 | 0.200 |
| 2012 | Polity IV - Democracy | 0.400 | 0.200 | 0.200 | 0.200 |
| 2011 | Polity IV - Democracy | 0.400 | 0.200 | 0.200 | 0.200 |
| 2010 | Polity IV - Democracy | 0.400 | 0.200 | 0.200 | 0.200 |
| 2015 | FI – Economic Freedom | 0.200 | 0.200 | 0.200 | 0.400 |
| 2014 | FI – Economic Freedom | 0.200 | 0.200 | 0.200 | 0.400 |
| 2013 | FI – Economic Freedom | 0.200 | 0.200 | 0.200 | 0.400 |
| 2012 | FI – Economic Freedom | 0.200 | 0.200 | 0.200 | 0.400 |
| 2011 | FI – Economic Freedom | 0.200 | 0.200 | 0.200 | 0.400 |
| 2010 | FI – Economic Freedom | 0.200 | 0.200 | 0.200 | 0.400 |
| 2015 | FI – Regulation | 0.200 | 0.200 | 0.200 | 0.400 |
| 2014 | FI – Regulation | 0.200 | 0.200 | 0.200 | 0.400 |
| 2013 | FI – Regulation | 0.203 | 0.200 | 0.200 | 0.397 |
| 2012 | FI – Regulation | 0.200 | 0.200 | 0.200 | 0.400 |
| 2011 | FI – Regulation | 0.200 | 0.200 | 0.200 | 0.400 |
| 2010 | FI – Regulation | 0.200 | 0.200 | 0.200 | 0.400 |

| Table S25. Combinations of components where the spanning is rejected for the dominated scenario when lower bound weight is set to 0.20 | | | | | |
| --- | --- | --- | --- | --- | --- |
| Year | Included governance proxy | Health | Education | Income | Governance |
| 2015 | CPI | 0.2 | 0.2 | 0.2 | 0.4 |
| 2014 | CPI | 0.2 | 0.2 | 0.2 | 0.4 |
| 2013 | CPI | 0.2 | 0.2 | 0.2 | 0.4 |
| 2012 | CPI | 0.2 | 0.2 | 0.2 | 0.4 |
| 2011 | CPI | 0.2 | 0.2 | 0.2 | 0.4 |
| 2010 | CPI | 0.2 | 0.2 | 0.2 | 0.4 |
| 2015 | HF - PR | 0.2 | 0.2 | 0.2 | 0.4 |
| 2014 | HF - PR | 0.2 | 0.2 | 0.2 | 0.4 |
| 2013 | HF - PR | 0.2 | 0.2 | 0.2 | 0.4 |
| 2012 | HF - PR | 0.2 | 0.2 | 0.2 | 0.4 |
| 2011 | HF - PR | 0.2 | 0.4 | 0.2 | 0.2 |
| 2010 | HF - PR | 0.2 | 0.2 | 0.2 | 0.4 |
| 2015 | FI - Judicial | 0.2 | 0.4 | 0.2 | 0.2 |
| 2014 | FI - Judicial | 0.2 | 0.4 | 0.2 | 0.2 |
| 2013 | FI - Judicial | 0.2 | 0.4 | 0.2 | 0.2 |
| 2012 | FI - Judicial | 0.2 | 0.4 | 0.2 | 0.2 |
| 2011 | FI - Judicial | 0.2 | 0.4 | 0.2 | 0.2 |
| 2010 | FI - Judicial | 0.2 | 0.4 | 0.2 | 0.2 |
| 2015 | FI - PR | 0.2 | 0.2 | 0.2 | 0.4 |
| 2014 | FI - PR | 0.2 | 0.2 | 0.2 | 0.4 |
| 2013 | FI - PR | 0.2 | 0.2 | 0.2 | 0.4 |
| 2012 | FI - PR | 0.2 | 0.2 | 0.2 | 0.4 |
| 2011 | FI - PR | 0.2 | 0.2 | 0.2 | 0.4 |
| 2010 | FI - PR | 0.2 | 0.2 | 0.2 | 0.4 |
| 2015 | FI - LSPR | 0.15 | 0.15 | 0.15 | 0.55 |
| 2014 | FI - LSPR | 0.15 | 0.15 | 0.15 | 0.55 |
| 2013 | FI - LSPR | 0.15 | 0.15 | 0.15 | 0.55 |
| 2012 | FI - LSPR | 0.15 | 0.15 | 0.15 | 0.55 |
| 2011 | FI - LSPR | 0.15 | 0.15 | 0.15 | 0.55 |
| 2010 | FI - LSPR | 0.15 | 0.15 | 0.15 | 0.55 |
| 2015 | FI - Bribes | 0.2 | 0.2 | 0.2 | 0.4 |
| 2014 | FI - Bribes | 0.2 | 0.2 | 0.2 | 0.4 |
| 2013 | FI - Bribes | 0.2 | 0.2 | 0.2 | 0.4 |
| 2012 | FI - Bribes | 0.2 | 0.2 | 0.2 | 0.4 |
| 2011 | FI - Bribes | 0.2 | 0.2 | 0.2 | 0.4 |
| 2010 | FI - Bribes | 0.2 | 0.2 | 0.2 | 0.4 |
| 2015 | FH - PR | 0.2 | 0.2 | 0.2 | 0.4 |
| 2014 | FH - PR | 0.2 | 0.2 | 0.2 | 0.4 |
| 2013 | FH - PR | 0.2 | 0.2 | 0.2 | 0.4 |
| 2012 | FH - PR | 0.2 | 0.2 | 0.2 | 0.4 |
| 2011 | FH - PR | 0.2 | 0.2 | 0.2 | 0.4 |
| 2015 | FH - CL | 0.2 | 0.2 | 0.2 | 0.4 |
| 2014 | FH - CL | 0.2 | 0.2 | 0.2 | 0.4 |
| 2013 | FH - CL | 0.2 | 0.2 | 0.2 | 0.4 |
| 2012 | FH - CL | 0.2 | 0.2 | 0.2 | 0.4 |
| 2010 | FH - CL | 0.2 | 0.2 | 0.2 | 0.4 |
| 2015 | FH - PRCL | 0.2 | 0.2 | 0.2 | 0.4 |
| 2014 | FH - PRCL | 0.2 | 0.2 | 0.2 | 0.4 |
| 2013 | FH - PRCL | 0.2 | 0.2 | 0.2 | 0.4 |
| 2012 | FH - PRCL | 0.2 | 0.2 | 0.2 | 0.4 |
| 2010 | FH - PRCL | 0.2 | 0.2 | 0.2 | 0.4 |
